# Projections of Extreme Temperature–Related Deaths in the US

**DOI:** 10.1001/jamanetworkopen.2024.34942

**Published:** 2024-09-20

**Authors:** Sameed Ahmed M. Khatana, Jonathan J. Szeto, Lauren A. Eberly, Ashwin S. Nathan, Jagadeesh Puvvula, Aimin Chen

**Affiliations:** 1 Division of Cardiovascular Medicine, Perelman School of Medicine, University of Pennsylvania, Philadelphia; 2 Penn Cardiovascular Outcomes, Quality, and Evaluative Research Center, Perelman School of Medicine, University of Pennsylvania, Philadelphia; 3 The Leonard Davis Institute of Health Economics, University of Pennsylvania, Philadelphia; 4 Perelman School of Medicine, University of Pennsylvania, Philadelphia; 5 Department of Biostatistics, Epidemiology and Informatics, Perelman School of Medicine, University of Pennsylvania, Philadelphia

## Abstract

**Importance:**

Extreme heat in the US is increasing due to climate change, while extreme cold is projected to decline. Understanding how extreme temperature along with demographic changes will affect population health is important for devising policies to mitigate the health outcome of climate change.

**Objective:**

To assess the burden of extreme temperature–related deaths in the contiguous US currently (2008-2019) and estimate the burden in the mid–21st century (2036-2065).

**Design, Setting, and Participants:**

This cross-sectional study used historical (1979-2000) daily mean temperatures to calculate monthly extreme heat (>97.5th percentile value) and extreme cold days (<2.5th percentile value) for all contiguous US counties for 2008 to 2019 (current period). Temperature projections from 20 climate models and county population projections were used to estimate extreme temperature–related deaths for 2036 to 2065 (mid–21st century period). Data were analyzed from November 2023 to July 2024.

**Exposure:**

Current monthly frequency of extreme heat days and projected mid–21st century frequency using 2 greenhouse gas emissions scenarios: Shared Socioeconomic Pathway (SSP)2-4.5, representing socioeconomic development with a lower emissions increase, and SSP5-8.5, representing higher emissions increase.

**Main Outcomes and Measures:**

Mean annual estimated number of extreme temperature–related excess deaths. Poisson regression model with county, month, and year fixed effects was used to estimate the association between extreme temperature and monthly all-cause mortality for older adults (aged ≥65 years) and younger adults (aged 18-64 years).

**Results:**

Across the contiguous US, extreme temperature days were associated with 8248.6 (95% CI, 4242.6-12 254.6) deaths annually in the current period and with 19 348.7 (95% CI, 11 388.7-27 308.6) projected deaths in the SSP2-4.5 scenario and 26 574.0 (95% CI, 15 408.0-37 740.1) in the SSP5-8.5 scenario. The mortality data included 30 924 133 decedents, of whom 15 573 699 were males (50.4%), with 6.3% of Hispanic ethnicity, 11.5% of non-Hispanic Black race, and 79.3% of non-Hispanic White race. Non-Hispanic Black adults (278.2%; 95% CI, 158.9%-397.5%) and Hispanic adults (537.5%; 95% CI, 261.6%-813.4%) were projected to have greater increases in extreme temperature–related deaths from the current period to the mid–21st century period compared with non-Hispanic White adults (70.8%; 95% CI, −5.8% to 147.3%).

**Conclusions and Relevance:**

This cross-sectional study found that extreme temperature–related deaths in the contiguous US were projected to increase substantially by mid–21st century, with certain populations, such as non-Hispanic Black and Hispanic adults, projected to disproportionately experience this increase. The results point to the need to mitigate the adverse outcome of extreme temperatures for population health.

## Key Points

**Question:**

Will anticipated variations in environmental temperatures and demographics in the US be associated with changes in temperature-related deaths in the coming decades?

**Findings:**

In this cross-sectional study of mortality and temperature data, extreme temperature–related deaths in the contiguous US were projected to increase substantially by mid–21st century (2036-2065) under different greenhouse gas emissions increase scenarios. A disproportionately large increase in extreme temperature–related deaths was projected for older, non-Hispanic Black, and Hispanic populations.

**Meaning:**

Findings of this study suggest that an increase in temperature-related deaths associated with climate change necessitates efforts to mitigate the adverse health outcome of extreme temperature.

## Teaser

This cross-sectional study estimates future temperature-related deaths associated with extreme heat and extreme cold temperatures modeled under 2 different greenhouse gas emission scenarios in the US.

## Introduction

In the US, due to anthropogenic greenhouse gas emissions, extreme heat has increased substantially and is projected to increase further in the coming decades.^1,2^ Concurrently, extreme cold episodes are declining.^3^ Understanding how these changes along with demographic changes will affect the burden of temperature-related mortality is crucial to informing climate change policy efforts to improve population health.

Human physiology has a narrow optimal temperature range.^4^ Extreme temperature exposure is associated with an increased risk of death, with cold temperatures associated with most of these deaths.^5,6,7^ In the US, certain subgroups, such as older adults and non-Hispanic Black adults, experience temperature-related adverse health outcomes to a disproportionate degree.^8,9,10,11^ As the climate warms, extreme heat will affect a larger portion of the country and for longer periods, which will coincide with an increase in the proportion of older adults and racial and ethnic minority populations.^12^ Additionally, different regions may experience warming to different degrees, and rural and urban areas may also be affected differentially.^13,14^ How these various factors will change the future burden of extreme temperature–related adverse health outcomes in different populations and regions is unclear. Understanding whether certain subgroups, based on age, sex, race and ethnicity, or area of residence, will experience disproportionally greater changes in extreme temperature–related adverse health outcomes may allow for more targeted interventions to mitigate such changes.

In this study, we aimed to assess the burden of extreme temperature–related mortality in the contiguous US currently (2008-2019) and in the mid–21st century (2036-2065). Specifically, we estimated how the number of excess deaths associated with extreme temperatures was projected to change from the current period to the coming decades based on 2 greenhouse gas emissions trajectories coupled with demographic scenarios in the country. These scenarios were Shared Socioeconomic Pathway (SSP)2-4.5, representing socioeconomic development with a lower emissions increase due to successful implementation of many currently proposed emissions control policies, and SSP5-8.5, representing fossil fuel–reliant socioeconomic development with a larger increase in emissions.^15,16^

## Methods

Restricted mortality data were obtained under agreement from the National Center for Health Statistics (NCHS) for this cross-sectional study. The University of Pennsylvania Institutional Review Board deemed this study exempt from ethics review and waived the informed consent requirement because publicly available, deidentified data were used. This study followed the Strengthening the Reporting of Observational Studies in Epidemiology (STROBE) reporting guideline.

### Current and Projected Extreme Temperature Days

Daily mean ambient temperature (mean of minimum and maximum temperature) was obtained from the Gridded Surface Meteorological (gridMET) dataset, which contains daily temperature at a high spatial resolution (4 km).^17,18^ The mean value of all centroids within a county’s boundaries was used to estimate county mean daily temperature. Analysis was conducted at the county level, the most granular geographic level available for the mortality data. A commonly used definition of extreme temperatures was over the 97.5th percentile value for hot days and under the 2.5th percentile value for cold days.^7,19,20^ The county-specific reference temperature was based on daily mean temperatures between 1979 and 2000. The monthly frequency of extremely hot or extremely cold days from the current period (2008-2019) was then determined.

Projected daily temperatures were obtained from the MACAv2-METDATA dataset, which contains 20 global climate models (GCMs) used in the Coupled Model Intercomparison Project Phase 5 statistically downscaled to a 4-km spatial resolution.^21^ To obtain stable estimates, we used 30-year (2036-2065) mean values to represent the mid–21st century period.^22^ The mean projected number of extreme temperature days (using the same thresholds for the current period) in each month was obtained for each GCM based on 2 potential greenhouse gas emissions trajectories: Representative Concentration Pathway (RCP) 4.5, representing an intermediate increase in emissions, and RCP 8.5, representing a larger increase in emissions.^23^ Additional details on these temperature datasets and RCPs are provided in the eMethods 1 to 3 in Supplement 1.

### Projected Population

County-level age, sex, and race and ethnicity–specific population data were obtained from previously published projections by Hauer for the mid–21st century period.^24^ These estimates were adjusted for different socioeconomic scenarios (SSPs) used in climate modeling. For this analysis, we used SSP2, which represents a middle-of-the-road socioeconomic scenario that assumes an intermediate level of challenges to climate change mitigation and adaptation, and SSP5, which represents fossil-fueled development.^23^ The mean projected annual population data for the mid–21st century period were used. Additional details on SSPs and the population projection methods are provided in eMethods 3 and 4 in Supplement 1. Based on plausible RCP and SSP pairings used for the Sixth Assessment Report of the Intergovernmental Panel on Climate Change, the SSP2 population projection was paired with the RCP 4.5 temperature projection, creating SSP2-4.5, and SSP5 was paired with RCP 8.5, creating SSP5-8.5.^25^

### Mortality Rates

Monthly, county-level, age-adjusted (standardized to the 2000 US Census), all-cause mortality rates among adults (aged ≥18 years) for the current period were used to estimate the association between extreme temperature and mortality. Mortality data included the age, sex, race and ethnicity of the deceased, and the month and county of death. Race and ethnicity data were obtained from death certificates, which have previously been shown to have greater than 90% agreement with self-reported race and ethnicity by Black, Hispanic, and White individuals.^26^ Population data were obtained from the US Census Bureau. To account for the instability of mortality rates from areas with small populations, we applied spatial empirical bayesian smoothing (eMethods 5 in Supplement 1).^27^

### Outcomes and Missing Data

The primary outcome was the mean annual estimated number of excess deaths associated with extreme temperature days during the current period and the mid–21st century period under the SSP2-4.5 and SSP5-8.5 scenarios. Secondary outcomes were region- and subgroup-specific excess deaths. Data sources and rates of missingness are listed in eMethods 6 in Supplement 1.

### Statistical Analysis

Summary measures of extreme temperature days and population were calculated. To estimate the association between monthly extreme temperature days and all-cause mortality rates in the current period, we fit a Poisson fixed-effects regression model with county, month, and year fixed effects. The monthly numbers of extreme heat and extreme cold days were included as separate variables. The model included restricted cubic splines of time-varying environmental, economic, demographic, and health care–related covariates, as listed in the eMethods 7 in Supplement 1, which have previously been associated with area-level mortality rates.^28,29,30,31^ The Poisson pseudo maximum likelihood estimation method with heteroskedasticity-robust SEs was used, as this approach has been shown to be robust to overdispersion.^32,33^ Based on the bayesian information criterion, a linear specification of extreme temperature days was used for the primary analysis (eTable 1 in Supplement 1). The temperature-mortality association was estimated for older adults (aged ≥65 years) and younger adults (aged, 18-64 years) separately, given the age-related heterogeneity in this association.^8,9^ Then, combining estimates for older and younger adults with county population levels, extreme temperature–related excess deaths in each county were calculated as the difference between estimated deaths with the observed number of extreme temperature days and deaths if no extreme temperature days had occurred. Using the regression coefficients and substituting the number of extreme temperature days and population with the projected values for each county under the 2 scenarios, we estimated the number of extreme temperature–related deaths in the mid–21st century period. Projections from each of the 20 GCMs were used separately, and the mean value of excess deaths across all GCMs was calculated.

Subgroup analyses categorized groups according to age, sex, race and ethnicity, US Census regions, and county metropolitan status (based on the 2013 NCHS Urban-Rural Classification Scheme for Counties). Sensitivity analyses were performed using a different threshold for extreme temperatures (>99th percentile and <1st percentile of extreme hot and cold temperatures, respectively), including lagged values of extreme temperature days, and using maximum daily heat index (incorporating relative humidity) for the definition of extreme heat.

Results are presented as means or medians with IQRs or 95% CIs. Two-sided *P* < .05 was considered statistically significant. Empirical Bayes smoothing was performed using the spdep package in R (R Project for Statistical Computing).^34^ All other analyses were performed using Stata 18 (StataCorp LLC). The Stata module ppmlhdfe was used to fit the Poisson fixed-effects model.^35^ Data were analyzed from November 2023 to July 2024.

## Results

Across all 3108 counties in the contiguous US, the daily mean (IQR) temperature threshold during the baseline period from 1979 to 2000 was 27.3 (25.3-28.8) °C for extreme heat and −6.38 (−11.46 to −0.9) °C for extreme cold. In the current period, the mean (IQR) annual number of extreme heat days was 13.3 (11.3-16.3) and the mean (IQR) annual number of extreme cold days was 8.2 (7.4-9.1) (Table 1). The projected mean (IQR) number of extreme heat days in the mid–21st century period was 40.7 (34.5-46.7) in the SSP2-4.5 scenario and 52.1 (44.7-59.5) in the SSP5-8.5 scenario. The projected mean (IQR) number of extreme cold days was 3.2 (2.6-4.0) in the SSP2-4.5 scenario and 2.6 (2.1-3.5) in the SSP5-8.5 scenario. Population and region-specific projections are listed in Table 1. The mortality data included 30 924 133 decedents, of whom 15 350 434 were females (49.6%), 15 573 699 were males (50.4%), with 6.3% of Hispanic ethnicity, 11.5% of non-Hispanic Black race, 79.3% of non-Hispanic White race, and 2.9% of non-Hispanic other race (including Asian, American Indian or Alaskan Native, and Pacific Islander).

**Table 1.** Current (2008-2019) and Projected (2036-2065) Mean Annual Number of Extreme Temperature Days and Population Across the Contiguous US and by Region^a^

	No. (IQR)		Projected change in mid–21st century vs current period, % (IQR)	SSP5-8.5 scenario, No. (IQR)^b^	Projected change in mid–21st century vs current period, % (IQR)
	Current period	SSP2-4.5 scenario^b^			
**Contiguous US**					
No. of days with extreme heat^c^	13.3 (11.3 to 16.3)	40.7 (34.5 to 46.7)	200.8 (164.4 to 241.9)	52.1 (44.7 to 59.5)	283.4 (234.5 to 337.7)
No. of days with extreme cold^d^	8.2 (7.4 to 9.1)	3.2 (2.6 to 4.0)	−61.3 (−69.6 to −49.5)	2.6 (2.1 to 3.5)	−67.7 (−75.9 to −56.4)
No. of adults (≥18 y)	19 524.3 (8337.4 to 50 287.1)	18 251.6 (7323.8 to 54 304.3)	−4.7 (−19.1 to 18.3)	20 747.3 (8350.5 to 62 265.9)	8.4 (−8.3 to 35.7)
No. of older adults (≥65 y)	4404.5 (2033.3 to 11 082.6)	5514.2 (2125.5 to 16 440.1)	19.0 (−4.6 to 71.9)	6267.2 (2409.3 to 18 951.4)	35.0 (7.7 to 99.8)
No. of younger adults (18- 64 y)	14 999.4 (6219.5 to 39 233.1)	12 791.4 (5182.6 to 38 127.6)	−13.2 (−26.6 to 7.5)	14 509.0 (5876.5 to 43 316.6)	−1.5 (−17.0 to 22.5)
**Midwest**					
No. of days with extreme heat^c^	11.5 (10.3 to 13.0)	35.2 (32.5 to 38.7)	207.1 (173.5 to 237.5)	45.0 (42.0 to 49.7)	294.6 (254.8 to 332.8)
No. of days with extreme cold^d^	8.4 (7.3 to 9.4)	2.6 (2.2 to 3.0)	−69.3 (−75.7 to −61.2)	2.14 (1.8 to 2.5)	−74.6 (−80.1 to −66.9)
No. of adults (≥18 y)	14 891.9 (6187.5 to 33 318.3)	13 162.6 (5254.8 to 32 168.2)	−9.2 (−19.5 to 4.7)	14 911.8 (5952.8 to 36747.1)	3.2 (−8.9 to 19.9)
No. of older adults (≥65 y)	3574.8 (16 17.3 to 7368.3)	4104.6 (1579.2 to 10 245.5)	8.5 (−7.9 to 38.3)	4651.2 (1785.2 to 11 692.9)	23.0 (4.0 to 57.9)
No. of younger adults (18- 64)	11 187.1 (4452.4 to 25 800.2)	9142.4 (3685.5 to 22 103.6)	−15.4 (−26.4 to −0.3)	10 285.7 (4183.2 to 24 960.0)	−3.8 (−16.8 to 13.8)
**Northeast**					
No. of days with extreme heat^c^	12.5 (11.6 to 13.8)	33.9 (32.5 to 35.0)	170.3 (144.5 to 189.6)	45.0 (42.6 to 46.3)	259.9 (220.3 to 284.7)
No. of days with extreme cold^d^	7.9 (7.1 to 8.5)	2.3 (2.0 to 2.5)	−69.4 (−74.4 to −66.6)	1.7 (1.5 to 1.9)	−77.1 (−81.4 to −74.7)
No. of adults (≥18 y)	77 389.8 (36168.3 to 222 544)	69 532.9 (29 319.0 to 244 384.7)	−11.5 (−20.3 to 5.9)	78 468.7 (33 469.9 to 280 485.5)	0.3 (−10.0 to 20.6)
No. of older adults (≥65 y)	17 054.7 (8730.0 to 45 553.1)	21 084.5 (9795.0 to 69 123.1)	20.7 (4.8 to 60.4)	23 733.3 (11 160.3 to 80 280.6)	36.6 (17.8 to 86.7)
No. of younger adults residents (18-64 y)	63 499.3 (27 800.8 to 176 091.9)	46 341.1 (20 130.8 to 165 251.7)	−21.1 (−29.4 to −6.1)	52 497.2 (22 743.9 to 186 745.3)	−9.9 (−20.3 to 7.0)
**South**					
No. of days with extreme heat^c^	15.0 (12.7 to 18.1)	46.2 (42.8 to 53.6)	211.1 (171.7 to 264.7)	58.9 (54.8 to 65.9)	294.7 (239.3 to 370.5)
No. of days with extreme cold^d^	8 (7.4 to 8.7)	4.0 (3.3 to 4.6)	−49.8 (−60.0 to −39.1)	3.5 (2.8 to 4.1)	−56.6 (−66.3 to −45.7)
No. of adults (≥18 y)	19 775.1 (10 227.1 to 46841.4)	19 050.1 (8954.4 to 52 377.1)	−2.2 (−19.6 to 26.7)	21 693.4 (10 158.9 to 60 075.1)	11.3 (−8.9 to 45.0)
No. of older adults (≥65 y)	4335.0 (2355.3 to 10 103.6)	5488.1 (2551.5 to 16 156.1)	25.6 (−3.8 to 87.1)	6236.8 (2884.2 to 18 529.6)	43.2 (8.5 to 117.2)
No. of younger adults (18-64 y)	15 182.9 (7722.5 to 36843.5)	13 449.1 (6230.2 to 36 870.0)	−11.7 (−26.3 to 13.1)	152 22.8 (7039.6 to 42 514.7)	0.3 (−16.7 to 29.6)
**West**					
No. of days with extreme heat^c^	14.8 (12.4 to 16.8)	38.8 (32.0 to 47.0)	164.3 (128.4 to 207.5)	48.1 (40.3 to 58.7)	232.4 (186.1 to 285.3)
No. of days with extreme cold^d^	8.9 (8.1 to 10.0)	3.3 (2.9 to 3.6)	−62.8 (−68.4 to −57.9)	2.5 (2.1 to 2.9)	−70.8 (−77.2 to −65.7)
No. of adults (≥18 y)	17 110.6 (6160.6 to 65 763.6)	17 881.2 (55 11.0 to 76 491.8)	8.9 (−14.2 to 34.5)	20 493.7 (6224.9 to 86 507.0)	25.2 (−2.9 to 54.2)
No. of older adults (≥65 y)	3873.0 (1482.1 to 13647.7)	5863.0 (1753.9 to 24 875.2)	48.8 (−1.0 to 113.3)	6736.2 (1992.5 to 28 729.0)	72.2 (11.9 to 149.7)
No. of younger adults (18-64 y)	13 097.1 (4526.3 to 50 098.5)	12 637.2 (3925.5 to 53 646.1)	−2.8 (−23.7 to 17.7)	14 463.4 (4442.0 to 61 485.4)	10.0 (−13.8 to 34.9)

Abbreviation: SSP, Shared Socioeconomic Pathway.

^a^ Mean annual numbers of extreme temperature days in each county for the current period (2008-2019) were based on data from gridMET and for the mid–21st century period (2036-2065) were based on mean of 20 global climate models from the MACAv2-METDATA dataset.

^b^ SSP2-4.5 refers to a middle-of-the-road scenario for socioeconomic changes and a lower increase in greenhouse gas emissions. SSP5-8.5 refers to a fossil-fueled development scenario for socioeconomic changes and a larger increase in greenhouse gas emissions.

^c^ Extreme heat is defined as any day with a mean temperature over 97.5th percentile of historical (1979-2000) daily values for the county.

^d^ Extreme cold is defined as any day with a mean temperature under 2.5th percentile of historical (1979-2000) daily values for the county.

**Figure 1.**
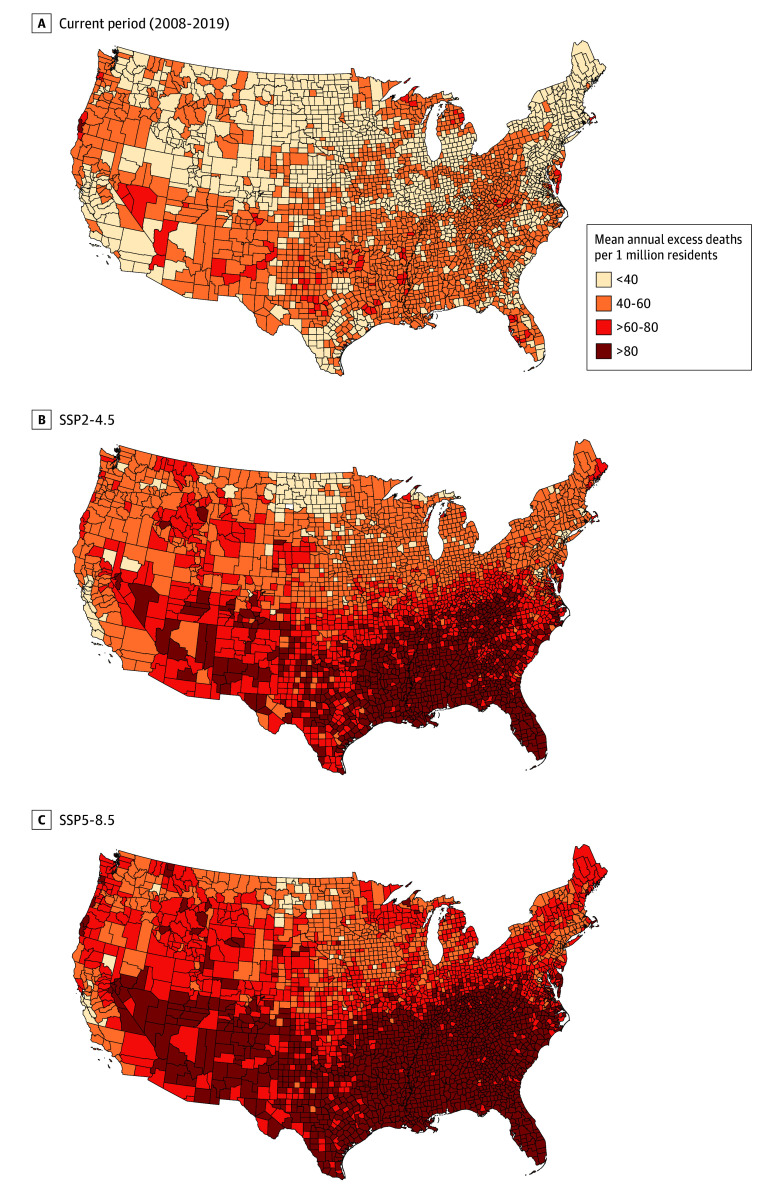
Estimated Mean Annual Extreme Temperature–Related Excess Deaths per 1 Million US Adults in the Current and Mid–21st Century Periods Excess deaths were estimated by calculating the difference between the number of predicted deaths in each county with all covariates at their observed value and the number of predicted deaths if there were no extreme heat days. For the current period (2008-2019), estimated excess deaths were based on Poisson fixed-effects model with monthly and annual covariates (described in eMethods 7 in Supplement 1). For the mid–21st century period (2036-2065), the number of extreme temperature days (hot and cold) and number of county population were replaced with projected values when calculating the difference while keeping the regression coefficients the same. Shared Socioeconomic Pathway (SSP)2-4.5 refers to a middle-of-the-road scenario for socioeconomic changes and a lower increase in greenhouse gas emissions. SSP5-8.5 refers to a fossil-fueled development scenario for socioeconomic changes and a larger increase in greenhouse gas emissions.

In the Poisson fixed-effects model, for the current period, 1 additional extreme heat day per month was associated with a 0.09% (95% CI, 0.04%-0.14%) higher monthly mortality rate among older adults and 0.17% (95% CI, 0.11%-0.23%) higher rate in younger adults (Table 2). Extreme cold days were associated with 0.26% (95% CI, 0.04%-0.48%) higher mortality rates among older adults and 0.27% (95% CI, 0.12%-0.41%) higher mortality rates among younger adults. The temperature-mortality associations for different subgroups and regions are shown in Table 2. The estimated mean annual number of deaths associated with extreme heat and extreme cold during the current period was 3137.0 (95% CI, 1867.7-4406.4) and 5111.6 (95% CI, 1417.3-8805.9), respectively (Table 3). The total number of extreme temperature–related deaths was 8248.6 (95% CI, 4242.6-12 254.6) or 35.4 (95% CI, 18.2-52.6) deaths per 1 million individuals per year (Figure 1; eTable 2 in Supplement 1).

**Table 2.** Age Group–Specific Percentage Change in Monthly Mortality Rate Associated With 1 Additional Extreme Heat Day in the Current Period (2008-2019)^a^

	Extreme heat days^b^				Extreme cold days^c^			
	Older adults^d^		Younger adults^e^		Older adults^d^		Younger adults^e^	
	% Change in monthly mortality (95% CI)	*P* value	% Change in monthly mortality (95% CI)	*P* value	% Change in monthly mortality (95% CI)	*P* value	% Change in monthly mortality (95% CI)	*P* value
All adults	0.09 (0.04 to 0.14)	.001	0.17 (0.11 to 0.23)	<.001	0.26 (0.04 to 0.48)	.02	0.27 (0.12 to 0.41)	<.001
Female	0.08 (0.03 to 0.14)	.002	0.13 (0.06 to 0.20)	<.001	0.20 (−0.05 to 0.46)	.12	0.26 (0.10 to 0.43)	.002
Male	0.09 (0.03 to 0.14)	.002	0.19 (0.12 to 0.26)	<.001	0.32 (0.13 to 0.51)	.001	0.27 (0.12 to 0.41)	<.001
US Census regions								
Midwest	0.09 (−0.02 to 0.19)	.12	0.28 (0.19 to 0.37)	<.001	0.03 (−0.42 to 0.48)	.91	0.14 (−0.10 to 0.39)	.25
Northeast	0.03 (−0.12 to 0.19)	.67	0.31 (0.18 to 0.44)	<.001	0.36 (0.02 to 0.70)	.04	0.27 (0.01 to 0.54)	.04
South	0.08 (0.03 to 0.13)	.001	0.15 (0.09 to 0.21)	<.001	0.40 (0.01 to 0.79)	.04	0.46 (0.19 to 0.74)	.001
West	0.08 (0.0 to 0.15)	.053	0.24 (0.10 to 0.38)	.001	0.22 (−0.06 to 0.51)	.13	0.19 (0.0 to 0.37)	.05
County metropolitan status^f^								
Metropolitan counties	0.10 (0.05 to 0.14)	<.001	0.17 (0.11 to 0.23)	<.001	0.25 (0.05 to 0.46)	.02	0.26 (0.12 to 0.41)	<.001
Nonmetropolitan counties	0.06 (−0.01 to 0.12)	.10	0.16 (0.06 to 0.26)	.002	0.29 (−0.05 to 0.64)	.10	0.28 (0.07 to 0.49)	.01

^a^ Based on Poisson fixed-effects model, with county, month, and year fixed effects as described in eMethods 7 in Supplement 1.

^b^ Extreme heat is defined as any day with a mean temperature over 97.5th percentile of historical (1979-2000) daily values for the county.

^c^ Extreme cold is defined as any day with a mean temperature under 2.5th percentile of historical (1979-2000) daily values for the county.

^d^ Aged 65 years or older.

^e^ Aged 18 to 64 years.

^f^ County metropolitan status was based on the 2013 National Center for Health Statistics Urban-Rural Classification Scheme for Counties.

**Table 3.** Estimated Mean Annual Number of Extreme Temperature–Related Deaths in the Current (2008-2019) and Mid–21st Century (2036-2065) Periods^a^

Extreme temperature^b^	Mean No. of extreme temperature–related deaths (95% CI)		
	Current period	SSP2-4.5 scenario^c^	SSP5-8.5 scenario^c^
All adults			
Extreme heat	3137.0 (1867.7 to 4406.4)	15 640.5 (8497.7 to 22 783.2)	22 936.4 (12 414 to 33 458.8)
Extreme cold	5111.6 (1417.3 to 8805.9)	3708.2 (850.5 to 6565.9)	3637.9 (828.5 to 6447.4)
Extreme temperatures combined	8248.6 (4242.6 to 12254.6)	19 348.7 (11 388.7 to 27 308.6)	26 574.0 (15 408.0 to 37 740.1)
Older adults (≥65 y)			
Extreme heat	1937.8 (815.9 to 3059.6)	11 637.6 (4864.9 to 18 410.4)	17 138.4 (7150.3 to 27 126.4)
Extreme cold	3962.0 (534.4 to 7389.7)	3185.8 (439.7 to 5931.8)	3132 (430.7 to 5833.2)
Extreme temperatures combined	5899.8 (2172.2 to 9627.4)	14 823.4 (7216.1 to 22 430.7)	20 270.1 (9615.2 to 30 925.1)
Younger adults (18-64 y)			
Extreme heat	1199.3 (714.9 to 1683.6)	4002.8 (2371.5 to 5634.2)	5798 (3430.7 to 8165.3)
Extreme cold	1149.6 (495.8 to 1803.4)	522.5 (226.6 to 818.3)	506 (219.3 to 792.7)
Extreme temperatures combined	2348.8 (1499.6 to 3198)	4525.3 (2839.0 to 6211.6)	6303.9 (3891.7 to 8716.1)
Sex			
Female			
Extreme heat	1269.5 (614.9 to 1924.1)	6548.301 (2926.918 to 10 169.68)	9560.667 (4259.43 to 14 861.9)
Extreme cold	1982.9 (−90.1 to 4055.9)	1413.013 (−166.7382 to 2992.765)	1378.126 (−164.9701 to 2921.222)
Extreme temperatures combined	3252.4 (1019.0 to 5485.7)	7961.314 (3855.337 to 12 067.29)	10 938.7 (5257.775 to 16 619.63)
Male			
Extreme heat	1836.3 (1102.8 to 2569.8)	8999.4 (4823.1 to 13 175.7)	13 263.0 (7066.7 to 19 459.4)
Extreme cold	3168.6 (1532 to 4805.2)	2363.5 (1081.8 to 3645.3)	2335.3 (1065.7 to 3604.9)
Extreme temperatures combined	5004.9 (3168.5 to 6841.3)	11 362.9 (6893.0 to 15 832.9)	15 598.2 (9169.5 to 22 026.9)
US Census regions			
Midwest			
Extreme heat	695.1 (148.8 to 1241.3)	2642.6 (346.1 to 4939.2)	3958.9 (508.8 to 7409)
Extreme cold	227.0 (−1540.1 to 1994.2)	76.3 (−668 to 820.7)	72.3 (−635.3 to 779.9)
Extreme temperatures combined	922.1 (−974.6 to 2818.8)	2719.0 (257.0 to 5180.9)	4031.2 (462.7 to 7599.6)
Northeast			
Extreme heat	451.3 (−199.6 to 1102.2)	1266.8 (−1118.2 to 3651.8)	1897.8 (−1686.1 to 5481.8)
Extreme cold	1116.7 (209.2 to 2024.3)	505.1 (77.0 to 933.1)	444.5 (67.6 to 821.4)
Extreme temperatures combined	1568.0 (390.4 to 2745.7)	1771.9 (−711.2 to 4255.0)	2342.4 (−1315.3 to 6000)
South			
Extreme heat	1274.9 (728.4 to 1821.4)	7740.5 (4044.1 to 11 436.9)	11 206.9 (5833.8 to 16 580)
Extreme cold	3043.9 (593.6 to 5494.1)	3221.5 (445.4 to 5997.5)	3243.3 (444.9 to 6041.8)
Extreme temperatures combined	4318.8 (1705.4 to 6932.1)	10962.0 (5872.1 to 16051.8)	14 450.2 (7865.2 to 21 035.3)
West			
Extreme heat	691.8 (249.3 to 1134.4)	3103.4 (812.2 to 5394.7)	4566.8 (1173.5 to 7960.1)
Extreme cold	965.5 (−162.1 to 2093.1)	685.8 (−143.8 to 1515.5)	673.2 (−142.5 to 1488.9)
Extreme temperatures combined	1657.3 (439.4 to 2875.3)	3789.2 (1349.8 to 6228.7)	5239.8 (1746.8 to 8732.8)
County metropolitan status^d^			
Metropolitan counties			
Extreme heat	2747.8 (1740.4 to 3755.2)	14788.5 (8714.2 to 20862.8)	21 716.7 (12 757.1 to 30 676.2)
Extreme cold	4092.2 (1347.7 to 6836.8)	3220.8 (906.9 to 5534.7)	3168.5 (887.0 to 5450.0)
Extreme temperatures combined	6840.0 (3838.9 to 9841.2)	18 009.3 (11 290.0 to 24 728.6)	24 884.9 (15 413.4 to 34 356.3)
Nonmetropolitan counties			
Extreme heat	408 (119.2 to 696.8)	1338.2 (291.4 to 2385.1)	1944.9 (417.9 to 3472)
Extreme cold	1033.1 (−32.6 to 2098.9)	464.1 (−24.9 to 953)	445.3 (−23.8 to 914.5)
Extreme temperatures combined	1441.1 (323.2 to 2559.1)	1802.3 (626.4 to 2978.2)	2390.3 (772.0 to 4008.5)

Abbreviation: SSP, Shared Socioeconomic Pathway.

^a^ Estimated excess deaths were based on Poisson fixed-effects model, with monthly and annual covariates from the 2008-2019 period (eMethods 7 in Supplement 1). Excess deaths were estimated by calculating the difference between the number of predicted deaths in each county with all covariates at their observed value and the number of predicted deaths if there were no extreme heat days. For the projected number of excess deaths in the mid–21st century period (2036-2065), the number of extreme temperature days (hot and cold) and number of county population were replaced with projected values when calculating the difference while keeping the regression coefficients the same.

^b^ Extreme heat is defined as any day with a mean temperature over 97.5th percentile of historical (1979-2000) daily values for the county. Extreme cold is defined as any day with a mean temperature under 2.5th percentile of historical (1979-2000) daily values for the county.

^c^ SSP2-4.5 refers to a middle-of-the-road scenario for socioeconomic changes and a lower increase in greenhouse gas emissions. SSP5-8.5 refers to a fossil-fueled development scenario for socioeconomic changes and a larger increase in greenhouse gas emissions.

^d^ County metropolitan status was based on the 2013 National Center for Health Statistics Urban-Rural Classification Scheme for Counties.

**Figure 2.**
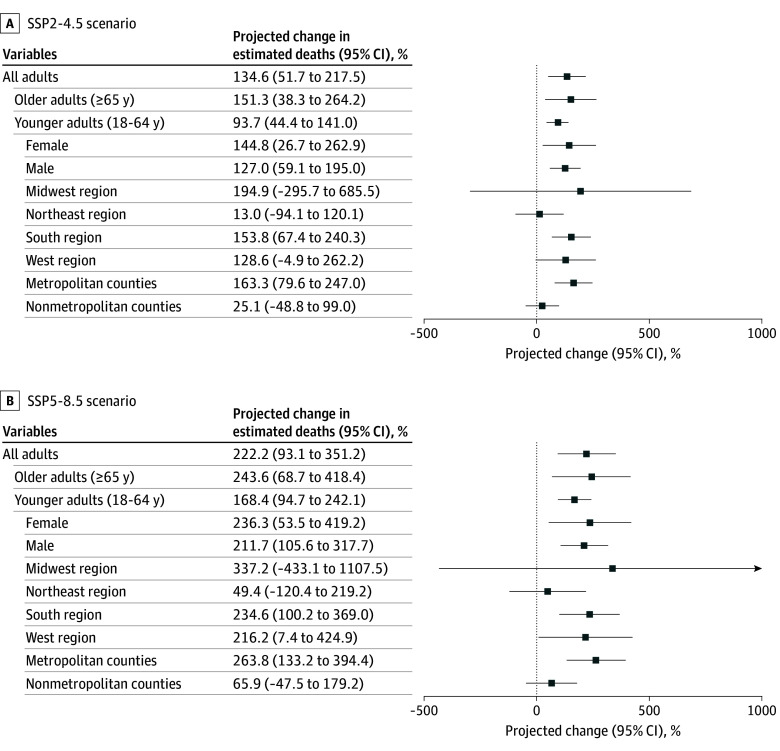
Projected Percentage Change in Estimated Mean Annual Extreme Temperature–Related Deaths From Current to Mid–21st Century Period Excess deaths were estimated by calculating the difference between the number of predicted deaths in each county with all covariates at their observed value and the number of predicted deaths if there were no extreme heat days. For the current period (2008-2019), estimated excess deaths were based on Poisson fixed-effects model with monthly and annual covariates (described in eMethods 7 in Supplement 1). For the mid–21st century period (2036-2065), the number of extreme temperature days (hot and cold) and number of county population were replaced with projected values when calculating the difference while keeping the regression coefficients the same. Shared Socioeconomic Pathway (SSP)2-4.5 refers to a middle-of-the-road scenario for socioeconomic changes and a lower increase in greenhouse gas emissions. SSP5-8.5 refers to a fossil-fueled development scenario for socioeconomic changes and a larger increase in greenhouse gas emissions. County metropolitan status was based on the 2013 National Center for Health Statistics Urban-Rural Classification Scheme for Counties.

In the mid–21st century period, the estimated annual number of extreme heat–related deaths was 15 640.5 (95% CI, 8497.7-22 783.2) in the SSP2-4.5 scenario and 22 936.4 (95% CI, 12 414.0-33 458.8) in the SSP5-8.5 scenario (Table 3). The estimated number of extreme cold–related deaths was 3708.2 (95% CI, 850.5-6565.9) in the SSP2-4.5 scenario and 3637.9 (95% CI, 828.5-6447.4) in the SSP5-8.5 scenario. The combined extreme temperature–related projected deaths in the mid–21st century period was 19 348.7 (95% CI, 11 388.7-27 308.6) for a 134.6% (95% CI, 51.7%-217.5%) change from the current period in the SSP2-4.5 scenario and was 26 574.0 (95% CI, 15 408.0-37 740.1) for a 222.2% (95% CI, 93.1%-351.2%) change in the SSP5-8.5 scenario (Figure 2; eTable 3 in Supplement 1). Per 1 million individuals, the projected number of extreme temperature–related deaths was 62.5 (95% CI, 36.8-88.3) in the SSP2-4.5 scenario and 74.9 (95% CI, 43.5-106.4) in the SSP5-8.5 scenario, representing a 76.6% (95% CI, 14.2%-139.1%) and 111.7% (95% CI, 26.9%-196.5%) change, respectively, compared with the current period (Figure 1; eTables 2 and 4 in Supplement 1).

In subgroup analyses for the SSP2-4.5 scenario, there was a projected increase in extreme temperature–related deaths of 151.3% (95% CI, 38.3%-264.2%) among older adults, 93.7% (95% CI, 44.4%-141.0%) among younger adults (93.7%; 95% CI, 44.4%-141.0%), 144.8% (95% CI, 26.7%-262.9%) among females, and 127.0% (95% CI, 59.1%-195.0%) among males (Figure 2; eTable 3 in Supplement 1). There was a projected increase in mortality of 537.5% (95% CI, 261.6%-813.4%) for Hispanic adults of any race, 278.2% (95% CI, 158.9%-397.5%) for non-Hispanic Black adults, 269.2% (95% CI, −66.4% to 604.7%) for non-Hispanic adults of other races, and 70.8% (95% CI, −5.8% to 147.3%) for non-Hispanic White adults compared with rates in the current period. The projected change was 163.3% (95% CI, 79.6%-247.0%) in metropolitan counties and 25.1% (95% CI, −48.8% to 99.0%) in nonmetropolitan counties. Projected changes in the SSP5-8.5 scenario and in each US Census region are provided in Figure 2 and eTable 3 in Supplement 1.

In the SSP2-4.5 scenario, the projected per capita change in extreme temperature–related deaths compared with the current period was 22.7% (95% CI, −32.5% to 77.8%) among older adults, 67.0% (95% CI, 25.1%-108.9%) among younger adults, 86.2% (95% CI, −3.6% to 176.0%) among females, 69.2% (95% CI, 18.5%-119.8%) among males, 164.5% (95% CI, 50.1%-279.0%) among Hispanic adults, 126.2% (95% CI, 54.9%-197.6%) among non-Hispanic Black adults, 71.3% (95% CI, −5.5% to 148.1%) among non-Hispanic adults of other races, and 65.4% (95% CI, −84.9% to 215.7%) among non-Hispanic White adults (eTable 4 in Supplement 1). There was a 89.5% (95% CI, 29.2%-149.7%) change in extreme temperature–related deaths in metropolitan counties and a 29.3% (95% CI, −47.1% to 105.7%) change in nonmetropolitan counties. Projected changes in per capita rates of extreme temperature–related deaths in the SSP5-8.5 scenario and each US Census region are shown in eTable 4 in Supplement 1. County-level estimates for the number of deaths per 1 million individuals for age and sex subgroups are provided in eFigures 1 to 4 in Supplement 1.

With an alternative extreme temperature definition (>99th percentile of historical daily mean temperatures for extreme heat and <1st percentile for extreme cold), the number of extreme temperature–related deaths were projected to change from 4897.3 (95% CI, 1920.8-7873.9) to 15 412.8 (95% CI, 8643.9-22 181.6) in the SSP2-4.5 scenario and 23 382.6 (95% CI, 12 980.5-33 784.7) in the SSP5-8.5 scenario. Including lagged monthly values of extreme temperature days and use of maximum daily heat index yielded projected estimates similar to those in the primary analyses (eTable 5 in Supplement 1).

## Discussion

In the current period (2008-2019), extreme temperatures were associated with 8248.6 deaths per year among adults living in the contiguous US. By the mid–21st century, after accounting for changes in both extreme heat and extreme cold, extreme temperature–related deaths were projected to increase to 19 348.7 deaths per year in the lower emissions increase scenario or 26 574.0 deaths in the higher emissions increase scenario. There was heterogeneity in the projected change among different subgroups, with the largest increases observed in older adults and non-Hispanic Black adults, Hispanic adults, and adults living in metropolitan counties.

Previous studies have documented the association between extreme temperature and increased mortality risk.^5,6,7,19^ To our knowledge, this study was the first to examine both extreme temperature–related deaths across all areas of the contiguous US rather than a selection of cities and projected changes across subgroups of age, sex, race and ethnicity, and regions. Although some studies have noted an increase in mortality at relatively mild temperatures, given the unclear biological mechanism and policy implications of deaths at milder temperatures, the focus of this analysis was on extreme temperatures.^5,6,36^ Some studies have suggested that in a warming planet, reductions in cold-related deaths may outweigh increases in heat-related deaths.^37,38^ However, this analysis noted that, although extreme cold–related deaths were projected to decrease due to a greater increase in heat-related deaths, extreme temperature–related deaths were projected to increase overall. A recent study using different methods, data from 106 US cities, and projections based on the SSP5-8.5 scenario estimated that extreme temperature–related deaths may increase 5-fold by the end of the 21st century with 3 °C of warming.^19^

A greater increase in temperature-related deaths was noted among older adults, which may reflect this population’s health-related vulnerability to extreme temperatures due to a higher prevalence of medical conditions, as well as the projected increase in the population of older adults by the mid–21st century. Additionally, non-Hispanic Black adults and Hispanic adults were projected to have a substantially greater increase in temperature-related deaths compared with non-Hispanic White adults. This estimate was in part attibutable to the projected increase in the proportion of individuals other than non-Hispanic White living in the US. However, non-Hispanic Black adults and Hispanic adults also had a greater increase in per capita rates, suggesting that the increase is not explained by population changes alone. Many individuals from ethnic and racial minority groups reside in neighborhoods that have lower access to air conditioning, a higher urban heat island effect, reduced green-space exposure, greater exposure to traffic-related air pollution, and a higher likelihood of winter power outages, which increase their vulnerability to extreme temperatures.^39,40,41,42^ Likely associated with factors such as the urban heat island effect, a greater increase in deaths was observed among metropolitan counties.

It is possible that communities may adapt to changes in extreme temperatures in the coming decades through measures such as increased adoption of air conditioning.^43^ Although this study did not account for the potential implications of such changes for the temperature-mortality association, even areas with nearly universal air conditioning access, such as the southern US, were found to have a high burden of extreme heat and temperature-related deaths.^44^ This finding suggests that there may be a limit to adaptation, especially through increasing air conditioning access. However, there is evidence that interventions, such as increasing urban tree cover, may be beneficial against extreme heat–related deaths.^45^ Evaluating the advantages and disadvantages of other interventions, such as heat action plans, is also important as policymakers devise strategies to mitigate the health outcomes of rising temperatures.

### Limitations

This study has some limitations. Due to the observational nature and ecological design of the study, causality was not established and individual-level inferences could not be made. As the primary exposure was the number of extreme temperature days per month, it was not possible to directly link a death to a particular extreme temperature day. The NCHS mortality dataset only provides the month of death; thus, a more temporally granular analysis was not possible. However, unlike studies using daily mortality data, the monthly data approach allowed for the inclusion of areas with smaller populations. Additionally, estimates of extreme temperature–related deaths in the current period were similar to those noted in studies using daily mortality data covering a substantial proportion of the US population.^19,46^ This study assumed the same temperature exposure across a given county and did not account for differences in temperature exposure within each county.

## Conclusions

The estimated number of deaths associated with extreme heat was projected to increase substantially in the contiguous US under 2 different greenhouse gas emissions increase scenarios. Certain populations, such as non-Hispanic Black and Hispanic adults, were projected to experience this increase disproportionately. Despite a decrease in extreme cold–related deaths, overall extreme temperature–related deaths were projected to more than double or triple depending on the emissions increase scenario analyzed. Along with efforts to reduce greenhouse gas emissions, efforts to mitigate the adverse outcomes of extreme temperatures for population health are needed.

## Notes

Supplement 1. **eMethods 1.** Temperature Data for Historical Baseline Period (1979-2000) and Current Period (2008-2019) **eMethods 2.** Global Climate Models and Projected Temperature Data for the Mid-century (2036-2065) Period **eMethods 3.** Representative Concentration Pathways (RCPs) and Shared Socioeconomic Pathways (SSPs) **eMethods 4.** County Population Projections **eMethods 5.** Spatial Empirical Bayes Smoothing **eMethods 6.** Data Sources and Data Missingness **eMethods 7.** Poisson Fixed Effects Regression Model **eTable 1.** Bayesian Information Criterion for Fixed Effects Models With Non-Linear Specifications for Extreme Temperature Days **eTable 2.** Estimated Mean Annual Number of Extreme Temperature Associated Deaths per 1 Million Individuals in Current (2008-2019) and Mid-century (2036-2065) Periods **eTable 3.** Projected Percent Change in Estimated Mean Annual Number of Extreme Temperature Related Deaths From Current (2008-2019) to Mid-century (2036-2065) Period **eTable 4.** Projected Percent Change in Estimated Mean Annual Number of Extreme Temperature Related Deaths per Capita From Current (2008-2019) to Mid-century (2036-2065) Period **eTable 5.** Estimated Excess Deaths Associated With Extreme Temperature Days in the Current (2008-2019) and Mid-century (2036-2065) Periods in the Contiguous United States Using Heat Index and Lagged Monthly Values **eFigure 1.** Estimated Mean Annual Extreme Temperature Associated Excess Deaths per 1 Million Adult Residents in the Current Period (2008-2019) and Mid-century (2036-2065) Projections for Older Adults (≥65 Years of Age) **eFigure 2.** Estimated Mean Annual Extreme Temperature Associated Excess Deaths per 1 Million Adult Residents in the Current Period (2008-2019) and Mid-century (2036-2065) Projections for Younger Adults (20-64 Years of Age) **eFigure 3.** Estimated Mean Annual Extreme Temperature Associated Excess Deaths per 1 Million Adult Residents in the Current Period (2008-2019) and Mid-century (2036-2065) Projections for Females **eFigure 4.** Estimated Mean Annual Extreme Temperature Associated Excess Deaths per 1 Million Adult Residents in the Current Period (2008-2019) and Mid-century (2036-2065) Projections for Males
eReferences

Supplement 2.
Data Sharing Statement
