# Supplementary material for: Projections of Extreme Temperature–Related Deaths in the US
Source: JAMA Netw Open. 2024 Sep 20;7(9):e2434942. doi: 10.1001/jamanetworkopen.2024.34942 (PMC11415784; doi:10.1001/jamanetworkopen.2024.34942)
Supplement: Supplement 1. — eMethods 1. Temperature Data for Historical Baseline Period (1979-2000) and Current Period (2008-2019) eMethods 2. Global Climate Models and Projected Temperature Data for the Mid-century (2036-2065) Period eMethods 3. Representative Concentration Pathways (RCPs) and Shared Socioeconomic Pathways (SSPs) eMethods 4. County Population Projections eMethods 5. Spatial Empirical Bayes Smoothing eMethods 6. Data Sources and Data Missingness eMethods 7. Poisson Fixed Effects Regression Model eTable 1. Bayesian Information Criterion for Fixed Effects Models With Non-Linear Specifications for Extreme Temperature Days eTable 2. Estimated Mean Annual Number of Extreme Temperature Associated Deaths per 1 Million Individuals in Current (2008-2019) and Mid-century (2036-2065) Periods eTable 3. Projected Percent Change in Estimated Mean Annual Number of Extreme Temperature Related Deaths From Current (2008-2019) to Mid-century (2036-2065) Period eTable 4. Projected Percent Change in Estimated Mean Annual Number of Extreme Temperature Related Deaths per Capita From Current (2008-2019) to Mid-century (2036-2065) Period eTable 5. Estimated Excess Deaths Associated With Extreme Temperature Days in the Current (2008-2019) and Mid-century (2036-2065) Periods in the Contiguous United States Using Heat Index and Lagged Monthly Values eFigure 1. Estimated Mean Annual Extreme Temperature Associated Excess Deaths per 1 Million Adult Residents in the Current Period (2008-2019) and Mid-century (2036-2065) Projections for Older Adults (≥65 Years of Age) eFigure 2. Estimated Mean Annual Extreme Temperature Associated Excess Deaths per 1 Million Adult Residents in the Current Period (2008-2019) and Mid-century (2036-2065) Projections for Younger Adults (20-64 Years of Age) eFigure 3. Estimated Mean Annual Extreme Temperature Associated Excess Deaths per 1 Million Adult Residents in the Current Period (2008-2019) and Mid-century (2036-2065) Projections for Females eFigure 4. Estimated Mean Annual [file jamanetwopen-e2434942-s001.pdf]

Supplemental Online Content

Khatana SAM, Szeto JJ, Eberly LA, Nathan AS, Puvvula J, Chen A. Projections of extreme temperature–related deaths in the US. *JAMA Netw Open*. 2024;7(9):e2434942. doi:10.1001/jamanetworkopen.2024.34942

eMethods 1. Temperature Data for Historical Baseline Period (1979-2000) and Current Period (2008-2019)

eMethods 2. Global Climate Models and Projected Temperature Data for the Mid-century (2036-2065) Period

eMethods 3. Representative Concentration Pathways (RCPs) and Shared Socioeconomic Pathways (SSPs)

eMethods 4. County Population Projections

eMethods 5. Spatial Empirical Bayes Smoothing

eMethods 6. Data Sources and Data Missingness

eMethods 7. Poisson Fixed Effects Regression Model

eTable 1. Bayesian Information Criterion for Fixed Effects Models With Non-Linear Specifications for Extreme Temperature Days

eTable 2. Estimated Mean Annual Number of Extreme Temperature Associated Deaths per 1 Million Individuals in Current (2008-2019) and Mid-century (2036-2065) Periods

eTable 3. Projected Percent Change in Estimated Mean Annual Number of Extreme Temperature Related Deaths From Current (2008-2019) to Mid-century (2036-2065) Period

eTable 4. Projected Percent Change in Estimated Mean Annual Number of Extreme Temperature Related Deaths per Capita From Current (2008-2019) to Mid-century (2036-2065) Period

eTable 5. Estimated Excess Deaths Associated With Extreme Temperature Days in the Current (2008-2019) and Mid-century (2036-2065) Periods in the Contiguous United States Using Heat Index and Lagged Monthly Values

eFigure 1. Estimated Mean Annual Extreme Temperature Associated Excess Deaths per 1 Million Adult Residents in the Current Period (2008-2019) and Mid-century (2036-2065) Projections for Older Adults (≥65 Years of Age)

eFigure 2. Estimated Mean Annual Extreme Temperature Associated Excess Deaths per 1 Million Adult Residents in the Current Period (2008-2019) and Mid-century (2036-2065) Projections for Younger Adults (20-64 Years of Age)

© 2024 Khatana SAM et al. *JAMA Network Open*

eFigure 3. Estimated Mean Annual Extreme Temperature Associated Excess Deaths per 1 Million Adult Residents in the Current Period (2008-2019) and Mid-century (2036-2065) Projections for Females

eFigure 4. Estimated Mean Annual Extreme Temperature Associated Excess Deaths per 1 Million Adult Residents in the Current Period (2008-2019) and Mid-century (2036-2065) Projections for Males

eReferences

This supplemental material has been provided by the authors to give readers additional information about their work.

© 2024 Khatana SAM et al. *JAMA Network Open*

eMethods 1 – Temperature data for historical baseline period (1979-2000) and current period (2008-2019)

Daily temperature data for the historical baseline period (1979-2000) and the current period (2008-2019) for each county in the contiguous United States (US) were obtained from gridMET.[1] Due to the insufficient density and lack of long-term continuous observations from weather station data, gridded meteorological datasets are necessary for processes requiring coverage in less populous areas of the country and over extended periods of time.[2] GridMET provides daily values of different surface meteorological variables at a spatially high-resolution (4 km). GridMET combines the desirable, high spatial attributes of the Parameter-elevation Regressions on Independent Slopes Model (PRISM) and high temporal attributes of the National Land Data Assimilation System Phase 2 (NLDAS-2) with bias corrects using climatically aided interpolation.[3] GridMET has been validated using observation date from >1500 Remote Automated Weather Stations (RAWS), 74 automated weather stations (AgriMet) from the US Bureau of Reclamation, and 44 observations from Washington State University's AgWeatherNet (AWN).

For the primary analysis, daily maximum and minimum temperatures and daily minimum relative humidity values for each year from 1979 to 2019 were obtained. GridMET netcdf data files were manipulated using Climate Data Operators (CDO) and netCDF Operators (NCO) software programs.[4,5] The daily mean value of maximum and minimum temperatures for each county were calculated by taking the mean of the values for each centroid inside a county's boundaries. The mean value of the daily maximum and minimum temperatures was used as the daily mean temperature. Maximum daily heat index was calculated using daily maximum temperature and daily minimum relative humidity values. Daily minimum relative humidity was used based on previous work suggesting that this provides the closest estimate to maximum heat index.[6] Heat index was calculated using the National Weather Service heat index equation.[7,8]

© 2024 Khatana SAM et al. *JAMA Network Open*

eMethods 2 – Global Climate Models and projected temperature data for the mid-century (2036-2065) period

Daily projected temperature data was obtained from 20 Global Climate Models (GCMs) used in the Coupled Model Intercomparison Project Phase 5 (CMIP5) statistically downscaled to a spatial resolution of 4 km using the Multivariate Adaptive Constructed Analogs (MACA) approach.[9] GCMs aim to represent major components of the climate (i.e. atmosphere, land surface, ocean, and sea ice) and the interactions between them, GCMs also attempt to mathematically predict future trajectories that the planet's climate may follow.[10] Using various inputs such as temperature, water vapor content, air pressure etc., GCMs model the interaction between the major climate components. GCMs divide the planet into 3D grids, with the resolution varying by model, but typically approximately 100 km^3.[11] In addition to modeling how climate components are changing over time, the results also model the exchange of energy and matter between neighboring grid cells. GCMs also contain a temporal component. GCMs are tested by "hind-casting" climate and weather conditions from previous time periods and assessing how this corresponds to observed values. GCMs can then be used to provide climate projections under different scenarios such as different radiative forcing (RF) scenarios (RF is the overall difference between incoming and outgoing energy across the planet).

To produce climate projections at a finer spatial resolution than produced by the original GCM output, the models need to be "down-scaled" using either a dynamical or statistical approach. While dynamical down-scaling uses the output of the GCMs in higher resolution Regional Climate Models (RCMs) to provide more detailed projections for a particular region, statistical downscaling uses a statistical approach to use the output of GCMs as predictor variables to predict the climate at a higher resolution.[12] For this analysis, we used statistically-downscaled GCMs produced using the MACA approach.[9] MACA uses a training dataset of observed values as the basis for removing historical biases and matching spatial patterns in GCM output. The MACAv2-METDATA uses the METDATA dataset for training and

© 2024 Khatana SAM et al. *JAMA Network Open*

downscales the GCM output to a 1/24° (~4 km) grid. The MACA approach has been validated using ERA-Interim reanalysis covering the western US from 1989 to 2008. The MACA approach was used to downscale the output from 20 GCMs that were used in the CMIP5 for the historical model forcings (1950-2005) and the two future greenhouse gas emissions trajectories based on two different Representative Concentration Pathways (RCPs) – RCP 4.5 and RCP 8.5 – from 2006 to 2100. The historical MACA output was found to identify extreme heat dates to a similar degree to observed values.[6] MACAv2-METDATA is also used by the US Forest Service for evaluating the impact of climate change on forests.[13]

The 20 GCMs included in MACAv2-METDATA are listed below:

**Global Climate Model**	**Ensemble**
bcc-csm1-1	r1i1p1
bcc-csm1-1-m	r1i1p1
BNU-ESM	r1i1p1
CanESM2	r1i1p1
CCSM4*	r6i1p1
CNRM-CM5	r1i1p1
CSIRO-Mk3-6-0	r1i1p1
GFDL-ESM2M	r1i1p1
GFDL-ESM2G	r1i1p1
HadGEM2-ES	r1i1p1
HadGEM2-CC	r1i1p1
inmcm4	r1i1p1
IPSL-CM5A-LR	r1i1p1

© 2024 Khatana SAM et al. *JAMA Network Open*

IPSL-CM5A-MR	r1i1p1
IPSL-CM5B-LR	r1i1p1
MIROC5	r1i1p1
MIROC-ESM	r1i1p1
MIROC-ESM-CHEM	r1i1p1
MRI-CGCM3	r1i1p1
NorESM1-M*	r1i1p1

* Projections from these GCMs were not used in the sensitivity analysis using heat index as projections for relative humidity are not available

After calculating the mean value of the projected relevant variable (temperature or relative humidity) in each county for each day between 2036 and 2065 for each of the 20 GCMs, the number of days in each month meeting extreme heat or extreme cold definitions, based on the mean temperature thresholds from the baseline historical period derived from gridMET, were identified. Then, for each GCM, the mean value of extreme heat or extreme cold days for each calendar month over the 30-year period was calculated. Taking the mean value over a 30-year period provides more stable estimates of the potential future climate under different scenarios.[6]

© 2024 Khatana SAM et al. *JAMA Network Open*

eMethods 3 – Representative Concentration Pathways (RCPs) and Shared Socioeconomic Pathways (SSPs)

A set of standard greenhouse gas emissions trajectories and socioeconomic development scenarios have been used in climate modeling to provide projections from different GCMs. Such projections are used to model how the climate may change, its impacts on human society, and the costs of efforts to mitigate the effects of climate change. Two complimentary modeling efforts have been undertaken by the research community – Representative Concentration Pathways (RCPs) focusing on climate projections and the Shared Socioeconomic Pathways (SSPs) focusing on projected trends in socioeconomic development.[14] The RCPs and SSPs have then been combined in a Scenario Matrix Architecture.[15]

Representative Concentration Pathways (RCPs)

RCPs attempt to model potential trajectories of atmospheric greenhouse gas concentrations under different possible emissions scenarios.[16] The RPCs were used for the Fifth Assessment Report of the Intergovernmental Panel on Climate Change (IPCC).[17] The RCPs were developed using Integrated Assessment Models (IAMs). IAMs are complex models that aim to integrate socioeconomic features and physical climate factors for climate modeling. Based on these IAMs, four RCPs were selected and are defined by their total radiative forcing (the overall difference between incoming and outgoing energy across the planet expressed in Watts per square meter) pathway and level by 2100. The four RCP scenarios are as follows:

RCP 2.6 – Represents a stringent mitigation pathway in which carbon dioxide (CO_2) emissions peak by 2020 and decline to zero by 2100. Atmospheric CO_2 concentrations peak around the middle of the 21st century and then begin declining. Global mean temperatures are projected to increase by 1.6°C (95% CI 0.9°C to 2.3°C) compared to the pre-industrial period.

RCP 4.5 – Represents an intermediate pathway in which greenhouse gas emissions peak near the middle of the 21st century and then begin declining. Atmospheric CO^2 concentrations continue to increase at current trends to the later part of the century, and then continue increase, but at a slower rate. Global mean temperatures are projected to increase by 2.4°C (95% CI 1.7°C to 3.2°C) compared to the pre-industrial period.

RCP 6.0 – Represents an intermediate scenario in which emissions increase rapidly through the later part of the century, followed by a dramatic decline. Atmospheric CO_2 concentrations continue to increase for the rest of the century but begin to increase at a slower rate near the end of the century. Global mean temperatures are projected to increase by 2.8°C (95% CI 2.0°C to 3.7°C) compared to the pre-industrial period.

RCP 8.5 – Represents a large greenhouse gas emissions increase scenario in which CO_2 emissions increase rapidly through the early and mid-century periods. Atmospheric CO_2 concentrations increase at an accelerating rate and continue to increase for an additional 100 years after 2100. Global mean temperatures are projected to increase by 4.3°C (95% CI 3.2°C to 5.4°C) compared to the pre-industrial period.

Shared Socioeconomic Pathways (SSPs)

SSPs are scenarios that cover a range of different socioeconomic changes that are likely to occur in the coming decades of the 21st century. SSPs were used for the Sixth Assessment Report of the IPCC.[18] The SSPs are narratives that describe a range of possible, and potentially likely, trajectories of socioeconomic developments that include a wide range of possible challenges to climate change mitigation and adaptation in human society.[15] These narratives are then used to project changes in different factors such as population, economic growth, urbanization, and technological advancement.[19] A summary of the different SSP narratives as described by Riahi et al. is as follows[15]:

© 2024 Khatana SAM et al. *JAMA Network Open*

“SSP1: Sustainability – Taking the Green Road (Low challenges to mitigation and adaptation)

The world shifts gradually, but pervasively, toward a more sustainable path, emphasizing more inclusive development that respects perceived environmental boundaries. Management of the global commons slowly improves, educational and health investments accelerate the demographic transition, and the emphasis on economic growth shifts toward a broader emphasis on human well-being. Driven by an increasing commitment to achieving development goals, inequality is reduced both across and within countries. Consumption is oriented toward low material growth and lower resource and energy intensity.

SSP2: Middle of the Road (Medium challenges to mitigation and adaptation)

The world follows a path in which social, economic, and technological trends do not shift markedly from historical patterns. Development and income growth proceeds unevenly, with some countries making relatively good progress while others fall short of expectations. Global and national institutions work toward but make slow progress in achieving sustainable development goals. Environmental systems experience degradation, although there are some improvements and overall the intensity of resource and energy use declines. Global population growth is moderate and levels off in the second half of the century. Income inequality persists or improves only slowly and challenges to reducing vulnerability to societal and environmental changes remain.

SSP3: Regional Rivalry – A Rocky Road (High challenges to mitigation and adaptation)

A resurgent nationalism, concerns about competitiveness and security, and regional conflicts push countries to increasingly focus on domestic or, at most, regional issues. Policies shift over time to become increasingly oriented toward national and regional security issues. Countries focus on achieving energy and food security goals within their own regions at the expense of broader-based development. Investments in education and technological development decline. Economic development is slow, consumption is material-intensive, and inequalities persist or worsen over time. Population growth is low

© 2024 Khatana SAM et al. *JAMA Network Open*

in industrialized and high in developing countries. A low international priority for addressing environmental concerns leads to strong environmental degradation in some regions.

SSP4: Inequality – A Road Divided (Low challenges to mitigation, high challenges to adaptation)

Highly unequal investments in human capital, combined with increasing disparities in economic opportunity and political power, lead to increasing inequalities and stratification both across and within countries. Over time, a gap widens between an internationally-connected society that contributes to knowledge- and capital-intensive sectors of the global economy, and a fragmented collection of lower-income, poorly educated societies that work in a labor intensive, low-tech economy. Social cohesion degrades and conflict and unrest become increasingly common. Technology development is high in the high-tech economy and sectors. The globally connected energy sector diversifies, with investments in both carbon-intensive fuels like coal and unconventional oil, but also low-carbon energy sources. Environmental policies focus on local issues around middle and high income areas.

SSP5: Fossil-fueled Development – Taking the Highway (High challenges to mitigation, low challenges to adaptation)

This world places increasing faith in competitive markets, innovation and participatory societies to produce rapid technological progress and development of human capital as the path to sustainable development. Global markets are increasingly integrated. There are also strong investments in health, education, and institutions to enhance human and social capital. At the same time, the push for economic and social development is coupled with the exploitation of abundant fossil fuel resources and the adoption of resource and energy intensive lifestyles around the world. All these factors lead to rapid growth of the global economy, while global population peaks and declines in the 21st century. Local environmental problems like air pollution are successfully managed. There is faith in the ability to effectively manage social and ecological systems, including by geo-engineering if necessary."

© 2024 Khatana SAM et al. *JAMA Network Open*

SSP1 and SSP5 represent scenarios in which rapid economic growth and increased investments in health and education occur. In SSP5 these investments and growth occur through fossil-fuel based development, while in SSP1 this occurs through more sustainable means. SSP3 and SSP4 are scenarios that envision lower economic growth and fewer improvements in social development. SSP2 represents a middle of the road scenario between the other scenarios.

The SSPs allow for projections of future population levels by translating their narratives into assumption of how fertility, mortality, migration and education for different regions will change.[20] SSP1 and SSP5 project the lowest global population levels at the end of the 21st century at around 7 billion. Population levels are projected to increase to 12.6 billion by 2100 under SSP3. Population projections for SSP2 and SSP4 are in between the other scenarios.

At baseline, the SSP narratives described above do not specifically include policies that can mitigate the impact of climate change. However, each SSP narrative describes different degrees of resistance and acceptance to, and need for, climate mitigation efforts that could allow for the achievement of emissions concentrations based on the different RCP trajectories. Although, in any of the SSP scenarios, with either more or less aggressive mitigation efforts, each of the different emissions concentration levels modeled in the RCP trajectories could be reached, certain RCP trajectories are more likely to occur under specific SSPs. For example, the RCP 8.5 trajectory is unlikely to be followed under SSP scenarios other than SSP5. For the Sixth Assessment Report of the IPCC, the following five SSP-RCP combination scenarios were used to assess a range of projected outcomes: SSP1-1.9, SSP1-2.6, SSP2-4.5, SSP3-7.0, and SSP5-8.5.[21]

For this analysis, we therefore used two commonly used coupled scenarios – SSP2-4.5 and SSP5-8.5. SSP2-4.5 represents scenario in which greenhouse gas emission concentrations increase to an intermediate degree with the enactment of many currently planned or ongoing mitigation measures.

© 2024 Khatana SAM et al. *JAMA Network Open*

SSP5-8.5 envisions a scenario with rapid fossil-fuel dependent development leading to a large increase in emissions.

© 2024 Khatana SAM et al. *JAMA Network Open*

eMethods 4 – County population projections

As described in eMethods 3, the SSP scenarios can project population levels for different regions based on the different narratives. Age, sex, race, and ethnicity specific county-level population projections for the mid-century (2036-2065) period estimated by Hauer were used in the analysis.[22] The Cohort-component method is the typical method for population projection, in which components of population change (i.e., fertility, mortality, migration) are projected separately for each birth cohort and then these components are used to project the population in the following year.[23] However, accurate data on such components is unavailable at the county level, especially for different subgroups. Hauer uses a common alternative to the Cohort-component method, a modification of the Hamilton-Perry method, which is a parsimonious method for creating population projections from multiple age-sex distributions using cohort-change ratios (CCRs).[24] As CCRs can become implausibly large and project explosive growth in cohorts with small populations, Hauer uses a blended model where county-race groups which are projected to grow utilize cohort-change differences (CCDs) instead, while county-race groups that are projected to decline utilize CCRs. Autoregressive integrated moving average (ARIMA) models were used to project the CCRs and CCDs. All age, sex, race, and county specific CCRs and CCDs were modeled in individual ARIMA models that populate the Leslie matrices (population projection matrices) to create the projected populations. After creating these projections, the age-structures were then adjusted for the five different SSP narratives described in eMethods 3 as each SSP narrative has been previously used to create age and sex-specific population projections.

The National Vital Statistics System (NVSS) U.S. Census Populations with Bridged Race Categories dataset was used to project the populations. This dataset bridges 31 race categories to four – Hispanic, non-Hispanic Black, non-Hispanic White, and Other. To evaluate the accuracy of the projections, age, sex, and race specific population projections for each county for the 2000 to 2015 period were made using a base

© 2024 Khatana SAM et al. *JAMA Network Open*

period of 1969 to 2000. These projections were then compared with the actual observed county populations for this time period, which showed a relatively small degree of bias.

© 2024 Khatana SAM et al. *JAMA Network Open*

eMethods 5 – Spatial empirical Bayes smoothing

Estimates of mortality rate from areas with small populations can be unstable as a small change in the absolute number of deaths leads to potentially large rate changes. This instability can lead to bias in the estimate of the mortality risk in such areas. To account for this, monthly mortality rates were smoothed using spatial empirical Bayes smoothing. This method combines the observed mortality rate with a reference rate and then calculates a weighted average of the two. The weights are directly proportional to the population of the geographical unit. Therefore, counties with small populations will have a greater adjustment in their mortality rates compared to counties with a larger population. After first specifying a prior distribution, a posterior distribution is obtained once the data is observed.

The standard approach for Bayesian smoothing is to specify a Poisson distribution for the observed counts (of deaths) and a Gamma prior distribution. This Poisson-Gamma mixture follows a negative binomial distribution. In an Empirical Bayes smoothing approach, parameters for the prior Gamma distribution are estimated from the observed data. The estimated prior rate can be considered the reference rate.

The empirical Bayes smoothed rate for a given county *i* is estimated using the following equation:

Smoothed $\text{Rate}_i = \omega_i \times \text{crude rate}_i + (1- \omega_i) \times \text{reference rate}_i$

where ω is a weight parameter calculated as follows:

$$\omega_i = \frac{\sigma^2}{(\sigma^2 + \mu/Population_i)}$$

where σ^2 and μ represent the variance and mean of the prior distribution and $Population_i$ refers to the population of county *i*.

μ is the reference mortality rate and is calculated as follows:

$$\sum_{i=1}^{i=n} Observed\ Deaths_i \,/ \sum_{i=1}^{i=n} Population_i$$

and the σ^2 as follows:

$$\frac{\sum_{i=1}^{1=n} Population_i (crude\ rate_i - \mu)^2}{\sum_{i=1}^{i=n} Population_i} - \frac{\mu}{\sum_{i=1}^{i=n} Population_i / n}$$

where n refers to the number of counties in the reference sample.

In spatial empirical Bayes, the mean and variance of the prior are estimated from a localized group of observations rather than the global sample (i.e. all US counties). In this analysis, we used all first-order neighboring counties as the reference group for each county. Smoothing was done for each month separately. For sub-group analyses, where a county had no deaths and all first-order neighboring counties also had zero deaths in a particular month, second-order neighbors were also included. If there were no deaths in first or second-order neighbors, then the mortality rate was considered to be zero.

© 2024 Khatana SAM et al. *JAMA Network Open*

eMethods 6 – Data sources and data missingness

Additional county-level publicly available data for this analysis were obtained from the following sources:

Monthly mean precipitation levels – Centers for Disease Control and Prevention's Environmental Public Health Tracking Program

Monthly mean fine particulate matter (PM2.5), and ozone concentrations – Environmental Protection Agency.

Monthly number of disaster declarations – Federal Emergency Management Agency

Monthly unemployment rate – Bureau for Labor Services

Total population, proportion of residents in different sub-groups based on age, gender, race and ethnicity, percentage of residents living in poverty, median household income, percentage of 18- to 64-year-old adults without health insurance, and county metropolitan status – United States Census Bureau

Percentage of county land covered by forest and the percentage of land developed (low, median and high intensity development) – Multi-Resolution Land Characteristics Consortium National Land Cover Database.

Number of primary care providers – Area Health Resources Files, Health Resources & Services Administration

Number of hospital beds – American Hospital Association annual survey

Percentage of adult residents with diabetes - United States Diabetes Surveillance System, Centers for Disease Control and Prevention

All mortality and heat data were available for all counties in the contiguous US for all included years. Covariate data were available for all counties and years except as follows:

The percentage of adult residents with diabetes in counties in the state of New Jersey were not available for 2019. Data from 2018 were used for both 2018 and 2019.

Percentage of county land covered by forest and the percentage of land developed is available for the following years: 2008, 2011, 2013, 2016, and 2019. As forest cover and development is not expected to change rapidly from year to year, we used values for 2008 in years 2008 and 2019, values for 2011 in years 2010 and 2011, values for 2013 in years 2012, 2013, and 2014, values for 2016 in years 2015, 2016, and 2017, and values for 2019 in years 2018 and 2019.

© 2024 Khatana SAM et al. *JAMA Network Open*

eMethods 7 – Poisson fixed effects regression model

The fixed effects, or within, estimator is a statistical model that can be used to analyze longitudinal or panel data. This modeling technique examines the association between change in the outcome with change in the predictor variable within each subject. Subject fixed effects (counties in this analysis) control for observed and un-observed time-invariant confounders. Time fixed effects account for secular time trends that are common for all subjects. The following Poisson fixed effects model was fit:

$$log(y_{imt}) = \beta_1 X_{imt} + a_i + \gamma_m + \zeta_t + \varepsilon_{imt} + log(population \times \ days\ in\ month_{imt})$$

Where y_{imt} is the number of age-adjusted, empirical Bayes smoothed deaths disease in county *i*, in month *m*, in year *t*, X_{imt} is a vector of time-varying independent variables, a_i is the county fixed effect, γ_m is the month fixed effect, ζ_t is the year fixed effect, ε_{imt} is the error term, and $\mathrm{population} \times \mathrm{days\ in\ month}_{itm}$ is the product of the annual county population and the number of days in month *m* (used as an offset term).

Two separate models – for elderly and non-elderly adults – were estimated simultaneously to allow for separate estimates of the association between extreme heat days and mortality for these two sub-groups.

The model included the following monthly variables:

the number of extreme heat days

the number of extreme cold days

mean precipitation levels

mean PM2.5 concentration

mean ozone concentrations

© 2024 Khatana SAM et al. *JAMA Network Open*

indicator for disaster declaration by the Federal Emergency Management Agency

unemployment rate

and annual variables:

poverty rate

inflation-adjusted median household income

percentage of county residents other than non-Hispanic White

percentage of county-residents who are female

percentage of adult residents with diabetes

percentage of non-elderly adults without health insurance

number of primary care providers per 100,000 residents

number of hospital beds per 100,000 residents

percentage of county land covered by forest

percentage of land developed

Except for the number of extreme heat days, all continuous variables are included as restricted cubic splines with 3 knots.

© 2024 Khatana SAM et al. *JAMA Network Open*

eTable 1 – Bayesian information criterion for fixed effects models with non-linear specifications for extreme temperature days*

Extreme temperature days per month†	Bayesian information criterion‡
Linear terms	3960833
Quadratic terms	3960862
Restricted cubic splines	3960927

* Other continuous covariates included as restricted cubic splines as eMethods 7. Includes all other covariates listed in eMethods 4

† Specification for the two extreme temperature (extreme heat and extreme cold) days per month variables in the fixed effects model

‡ Lower value indicates better model fit

© 2024 Khatana SAM et al. *JAMA Network Open*

eTable 2 – Estimated mean annual number of extreme temperature associated deaths per 1 million individuals in current (2008-2019) and mid-century (2036-2065) periods*

	Current period n (95% CI)	SSP2-4.5† n (95% CI)	SSP5-8.5† n (95% CI)
All adults	35.4 (18.2, 52.6)	62.5 (36.8, 88.3)	74.9 (43.5, 106.4)
**Sub-groups**			
Older adults (65 years and older)	130.0 (47.9, 212.1)	159.4 (77.6, 241.3)	188.2 (89.3, 287.1)
Younger adults (18-64 years old)	12.5 (8.0, 17.0)	20.9 (13.1, 28.69875)	25.5 (15.8, 35.3)
Female	27.1 (8.5, 45.7)	50.5 (24.4, 76.5)	60.7 (29.2, 92.3)
Male	44.3 (28.0, 60.5)	74.9 (45.4, 104.4)	89.4 (52.6, 126.2)
Hispanic adults of any race	22.6 (13.8, 31.4)	59.8 (26.1, 93.6)	69.9 (27.7, 112.1)
Non-Hispanic Black adults	50.5 (31.4, 69.6)	114.2 (73.1, 155.4)	135.7 (85.0, 186.5)
Non-Hispanic white adults	36.7 (14.2, 59.2)	62.8 (30.6, 95.0)	75.1 (35.8, 114.3)
Non-Hispanic adults of other races ‡	15.9 (7.6, 24.1)	26.3 (-5.2, 57.7)	30.6 (-10.1, 71.3)
**US Census regions**			
Midwest	18.5 (-19.5, 56.5)	49.0 (4.6, 93.3)	63.5 (7.3, 119.8)
Northeast	37.2 (9.3, 65.1)	37.6 (-15.1, 90.4)	43.6 (-24.5, 111.7)
South	49.2 (19.4, 79.0)	84.6 (45.3, 123.9)	97.1 (52.9, 141.4)
West	31.2 (8.3, 54.1)	49.0 (17.5, 80.6)	59.1 (19.7, 98.5)
**County metropolitan status §**			
Metropolitan counties	34.4 (19.3, 49.5)	65.2 (40.8, 89.5)	78.5 (48.6, 108.4)
Non-metropolitan counties	42.2 (9.5, 75.0)	54.6 (19.0, 90.2)	63.6 (20.5, 106.7)

* Estimated excess deaths based on Poisson fixed effects model with monthly and annual covariates from the 2008-2019 period (eMethods 7). Excess deaths were then estimated by calculating the difference between the number of predicted deaths in each county with all covariates at their observed value and the number of predicted deaths if there were no extreme heat days. For the projected number of excess deaths in the mid-century period, the number of extreme temperature days (hot and cold) and county population were replaced with projected values when calculating the difference while keeping the regression coefficients the same. Extreme heat defined as any day with mean temperature >97.5th percentile of historical (1979-2000) daily values for the county. Extreme cold defined as any day with mean temperature <2.5th percentile of

© 2024 Khatana SAM et al. *JAMA Network Open*

historical (1979-2000) daily values for the county. Current and projected population used to calculate the number of deaths per 1 million individuals.

† SSP - Shared Socioeconomic Pathways. RCP - Representative Concentration Pathway. SSP2-4.5 refers to a “Middle of the road” scenario for socio-economic changes and a lower increase in greenhouse gas emissions. SSP5-8.5 refers to a “Fossil-Fueled Development” scenario for socio-economic changes and a larger increase in greenhouse gas emissions.

‡ Subgroup consists of individuals identified as non-Hispanic ethnicity and any of the following race groups: American Indian (includes Aleuts and Eskimos), Chinese, Japanese, Hawaiian (includes Part-Hawaiian), Filipino, Asian Indian, Korean, Samoan, Vietnamese, Guamanian, Other Asian or Pacific Islander, Combined other Asian or Pacific Islander

§ County metropolitan status based on the 2013 National Center for Health Statistics Urban-Rural Classification Scheme

© 2024 Khatana SAM et al. *JAMA Network Open*

eTable 3 – Projected percent change in estimated mean annual number of extreme temperature related deaths from current (2008-2019) to mid-century (2036-2065) period*

	Extreme temperature†	SSP2-4.5 ‡<br>% (95% CI)	SSP5-8.5 ‡<br>% (95% CI)
All adults	Extreme heat	398.6 (355.6, 441.6)	631.1 (566.2, 696.1)
	Extreme cold	-27.5 (-32.7, -22.2)	-28.8 (-34.1, -23.6)
	Extreme temperatures combined	134.6 (51.7, 217.5)	222.2 (93.1, 351.2)
**Sub-groups**			
Older adults (65 years and older)	Extreme heat	500.6 (493.0, 508.1)	784.4 (773.0, 795.9)
	Extreme cold	-19.6 (-20.8, -18.4)	-20.9 (-22.2, -19.7)
	Extreme temperatures combined	151.3 (38.3, 264.2)	243.6 (68.7, 418.4)
Younger adults (18-64 years old)	Extreme heat	233.8 (230.0, 238.0)	383.5 (378.0, 388.9)
	Extreme cold	-54.6 (-55.3, -53.8)	-56.0 (-56.9, -55.1)
	Extreme temperatures combined	93.7 (44.4, 141.0)	168.4 (94.7, 242.1)
Female	Extreme heat	415.8 (378.2, 453.4)	653.1 (596.9, 709.3)
	Extreme cold	-28.7 (-35.4, -22.1)	-30.5 (-37.1, -23.9)
	Extreme temperatures combined	144.8 (26.7, 262.9)	236.3 (53.5, 419.2)
Male	Extreme heat	390.1 (336.9, 443.3)	622.3 (540.9, 703.6)
	Extreme cold	-25.4 (-29.9, -20.9)	-26.3 (-30.9, -21.7)
	Extreme temperatures combined	127.0 (59.1, 195.0)	211.7 (105.6, 317.7)
Hispanic adults of any race	Extreme heat	1260.8 (800, 1721.7)	2071.4 (1331.5, 2811.3)
	Extreme cold	127.5 (88.7, 166.2)	149.7 (106.4, 193)
	Extreme temperatures combined	537.5 (261.6, 813.4)	844.9 (387.2, 1302.6)
Non-Hispanic Black adults	Extreme heat	749.9 (603.9, 895.9)	1250.7 (1016.1, 1485.2)
	Extreme cold	25.8 (7.3, 44.4)	35.5 (15.3, 55.8)
	Extreme temperatures combined	278.2 (158.9, 397.5)	459.0 (262.5, 655.5)
Non-Hispanic white adults	Extreme heat	264.1 (221.9, 306.3)	463.6 (396.9, 530.4)
	Extreme cold	-46.2 (-49.8, -42.5)	-45 (-48.8, -41.2)
	Extreme temperatures combined	70.8 (-5.8, 147.3)	146.7 (21.0, 272.4)

© 2024 Khatana SAM et al. *JAMA Network Open*

Non-Hispanic adults of other races §	Extreme heat	858 (56.3, 1659.6)	1459.9 (130.2, 2789.6)
	Extreme cold	48.1 (18, 78.1)	58.8 (25.8, 91.8)
	Extreme temperatures combined	269.2 (-66.4, 604.7)	441.3 (-128.3, 1011)
**US Census regions**			
Midwest	Extreme heat	280.2 (204.4, 356)	469.6 (353.2, 586)
	Extreme cold	-66.4 (-134.2, 1.5)	-68.2 (-133.6, -2.7)
	Extreme temperatures combined	194.9 (-295.7, 685.5)	337.2 (-433.1, 1107.5)
Northeast	Extreme heat	180.7 (47.1, 314.3)	320.5 (117.8, 523.2)
	Extreme cold	-54.8 (-58.3, -51.2)	-60.2 (-63.4, -57)
	Extreme temperatures combined	13.0 (-94.1, 120.1)	49.4 (-120.4, 219.2)
South	Extreme heat	507.1 (450, 564.2)	779 (694.1, 863.9)
	Extreme cold	5.8 (-3.4, 15.1)	6.6 (-2.9, 16)
	Extreme temperatures combined	153.8 (67.4, 240.3)	234.6 (100.2, 369)
West	Extreme heat	348.6 (263.9, 433.2)	560.1 (431.4, 688.8)
	Extreme cold	-29 (-35.7, -22.3)	-30.3 (-37.4, -23.2)
	Extreme temperatures combined	128.6 (-4.9, 262.2)	216.2 (7.4, 424.9)
**County metropolitan status ‖**			
Metropolitan counties	Extreme heat	438.2 (393.4, 483)	690.3 (622.7, 758)
	Extreme cold	-21.3 (-27.2, -15.4)	-22.6 (-28.5, -16.6)
	Extreme temperatures combined	163.3 (79.6, 247)	263.8 (133.2, 394.4)
Non-metropolitan counties	Extreme heat	228 (192.3, 263.7)	376.7 (323.2, 430.1)
	Extreme cold	-55.1 (-56.8, -53.3)	-56.9 (-58.6, -55.2)
	Extreme temperatures combined	25.1 (-48.8, 99)	65.9 (-47.5, 179.2)

* Estimated excess deaths based on Poisson fixed effects model with monthly and annual covariates from the 2008-2019 period (eMethods 7). Excess deaths were then estimated by calculating the difference between the number of predicted deaths in each county with all covariates at their observed value and the number of predicted deaths if there were no extreme temperature days. For the projected number of excess deaths in the mid-century period, the number of extreme heat days and county population were replaced with projected values when calculating the difference while keeping the regression coefficients the same.

© 2024 Khatana SAM et al. *JAMA Network Open*

†Extreme heat defined as any day with mean temperature >97.5th percentile of historical (1979-2000) daily values for the county. Extreme cold defined as any day with mean temperature <2.5th percentile of historical (1979-2000) daily values for the county

‡ SSP - Shared Socioeconomic Pathways. RCP - Representative Concentration Pathway. SSP2-4.5 refers to a "Middle of the road" scenario for socio-economic changes and a lower increase in greenhouse gas emissions. SSP5-8.5 refers to a "Fossil-Fueled Development" scenario for socio-economic changes and a larger increase in greenhouse gas emissions.

§ Subgroup consists of individuals identified as non-Hispanic ethnicity and any of the following race groups: American Indian (includes Aleuts and Eskimos), Chinese, Japanese, Hawaiian (includes Part-Hawaiian), Filipino, Asian Indian, Korean, Samoan, Vietnamese, Guamanian, Other Asian or Pacific Islander, Combined other Asian or Pacific Islander

‖ County metropolitan status based on the 2013 National Center for Health Statistics Urban-Rural Classification Scheme

eTable 4 – Projected percent change in estimated mean annual number of extreme temperature related deaths per capita from current (2008-2019) to mid-century (2036-2065) period*

	SSP2-4.5 ‡<br>% (95% CI)	SSP5-8.5 ‡<br>% (95% CI)
All adults	76.6 (14.2, 139.1)	111.7 (26.9, 196.5)
**Sub-groups**		
Older adults (65 years and older)	22.7 (-32.5, 77.8)	44.8 (-28.9, 118.5)
Younger adults (18-64 years old)	67.0 (25.1, 108.9)	104.0 (47.9, 160.0)
Female	86.2 (-3.6, 176.0)	124.0 (2.2, 245.8)
Male	69.2 (18.5, 119.8)	102.0 (33.2, 170.7)
Hispanic adults of any race	164.5 (50.1, 279.0)	209.0 (59.3, 358.7)
Non-Hispanic Black adults	126.2 (54.9, 197.6)	168.8 (74.3, 263.3)
Non-Hispanic white adults	71.3 (-5.5, 148.1)	104.7 (0.4, 209.0)
Non-Hispanic adults of other races ‡	65.4 (-84.9, 215.7)	92.7 (-110.1, 295.5)
**US Census regions**		
Midwest	165.0 (-275.9, 605.9)	243.8 (-361.9, 849.5)
Northeast	1.3 (-94.7, 97.2)	17.3 (-116.0, 150.6)
South	72.0 (13.4, 130.6)	97.5 (18.2, 176.8)
West	57.3 (-34.6, 149.1)	89.5 (-35.7, 214.6)
**County metropolitan status §**		
Metropolitan counties	89.5 (29.2, 149.7)	128.2 (46.3, 210.2)
Non-metropolitan counties	29.3 (-47.1, 105.7)	50.6 (-52.4, 153.5)

© 2024 Khatana SAM et al. *JAMA Network Open*

* Estimated excess deaths based on Poisson fixed effects model with monthly and annual covariates from the 2008-2019 period (eMethods 7). Excess deaths were then estimated by calculating the difference between the number of predicted deaths in each county with all covariates at their observed value and the number of predicted deaths if there were no extreme heat days. For the projected number of excess deaths in the mid-century period, the number of extreme temperature days (hot and cold) and county population were replaced with projected values when calculating the difference while keeping the regression coefficients the same. Extreme heat defined as any day with mean temperature >97.5th percentile of historical (1979-2000) daily values for the county. Extreme cold defined as any day with mean temperature <2.5th percentile of historical (1979-2000) daily values for the county

† SSP - Shared Socioeconomic Pathways. RCP - Representative Concentration Pathway. SSP2-4.5 refers to a "Middle of the road" scenario for socio-economic changes and a lower increase in greenhouse gas emissions. SSP5-8.5 refers to a "Fossil-Fueled Development" scenario for socio-economic changes and a larger increase in greenhouse gas emissions.

‡ Subgroup consists of individuals identified as non-Hispanic ethnicity and any of the following race groups: American Indian (includes Aleuts and Eskimos), Chinese, Japanese, Hawaiian (includes Part-Hawaiian), Filipino, Asian Indian, Korean, Samoan, Vietnamese, Guamanian, Other Asian or Pacific Islander, Combined other Asian or Pacific Islander

§ County metropolitan status based on the 2013 National Center for Health Statistics Urban-Rural Classification Scheme

© 2024 Khatana SAM et al. *JAMA Network Open*

eTable 5 – Estimated excess deaths associated with extreme temperature days in the current (2008-2019) and mid-century (2036-2065) periods in the contiguous United States using heat index and lagged monthly values*

	**Current period**	**SSP2-4.5†**		**SSP5-8.5†**	
**Model**	**Estimated excess deaths number of deaths, (95% CI)**	**Estimated excess deaths number of deaths, (95% CI)**	**Percent change compared to current period %, (95% CI)**	**Estimated excess deaths number of deaths, (95% CI)**	**Percent change compared to current period %, (95% CI)**
Using maximum daily heat index values to define extreme heat ‡	7460.7 (3538.8, 11382.7)	19728.5 (11012.7, 28444.2)	164.4 (54.5, 274.3)	27269.6 (14904.7, 39634.5)	265.5 (96.2, 434.8)
Including lagged monthly values of extreme heat and extreme cold days §	12002.1 (3913.6, 20092.3)	23775.7 (9612.1, 37939.3)	98.1 (38.0, 158.1)	26681.2 (11990.3, 41372.2)	122.3 (39.1, 205.5)

* Estimated excess deaths based on Poisson fixed effects model with monthly and annual covariates from the 2008-2019 period (eMethods 7). Excess deaths were then estimated by calculating the difference between the number of predicted deaths in each county with all covariates at their observed value and the number of predicted deaths if there were no extreme heat days. For the projected number of excess deaths in the mid-century period, the number of extreme heat days and county population were replaced with projected values when calculating the difference while keeping the regression coefficients the same.

† SSP - Shared Socioeconomic Pathways. SSP2-4.5 refers to a “Middle of the road” scenario for socio-economic changes and an intermediate increase in greenhouse gas emissions trajectory. SSP5-8.5 refers to a “Fossil-Fueled Development” scenario for socio-economic changes and a large increase in greenhouse gas emissions trajectory.

‡ Extreme heat defined if daily maximum heat index >97.5th percentile of daily values from historical period (1979-2000)

§ Model includes monthly extreme temperature (extreme heat and cold) days as well as first lag of monthly values

© 2024 Khatana SAM et al. *JAMA Network Open*

eFigure 1 – Estimated mean annual extreme temperature associated excess deaths per 1 million adult residents in the current period (2008-2019) and mid-century (2036-2065) projections for older adults (≥65 years of age)*

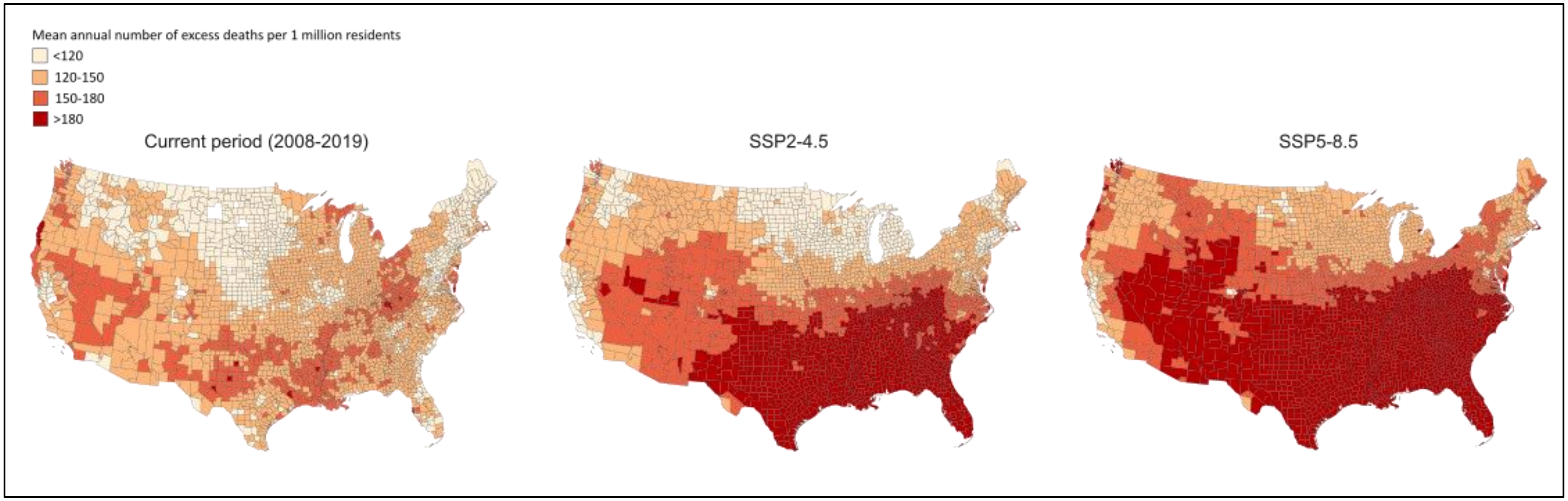

* Extreme heat defined as any day with mean temperature >97.5th percentile of historical (1979-2000) daily values for the county. Extreme cold defined as any day with mean temperature <2.5th percentile of historical (1979-2000) daily values for the county.

Estimated excess deaths based on Poisson fixed effects model with monthly and annual covariates from the 2008-2019 period (eMethods 7). Excess deaths were then estimated by calculating the difference between the number of predicted deaths in each county with all covariates at their observed value and the number of predicted deaths if there were no extreme heat days. For the projected number of excess deaths in the mid-century period, the number of extreme temperature days (hot and cold) and county population were replaced with projected values when calculating the difference while keeping the regression coefficients the same.

SSP - Shared Socioeconomic Pathways. RCP - Representative Concentration Pathway. SSP2-4.5 refers to a “Middle of the road” scenario for socio-economic changes and a lower increase in greenhouse gas emissions. SSP5-8.5 refers to a “Fossil-Fueled Development” scenario for socio-economic changes and a larger increase in greenhouse gas emissions.

© 2024 Khatana SAM et al. *JAMA Network Open*

eFigure 2 – Estimated mean annual extreme temperature associated excess deaths per 1 million adult residents in the current period (2008-2019) and mid-century (2036-2065) projections for younger adults (20-64 years of age)*

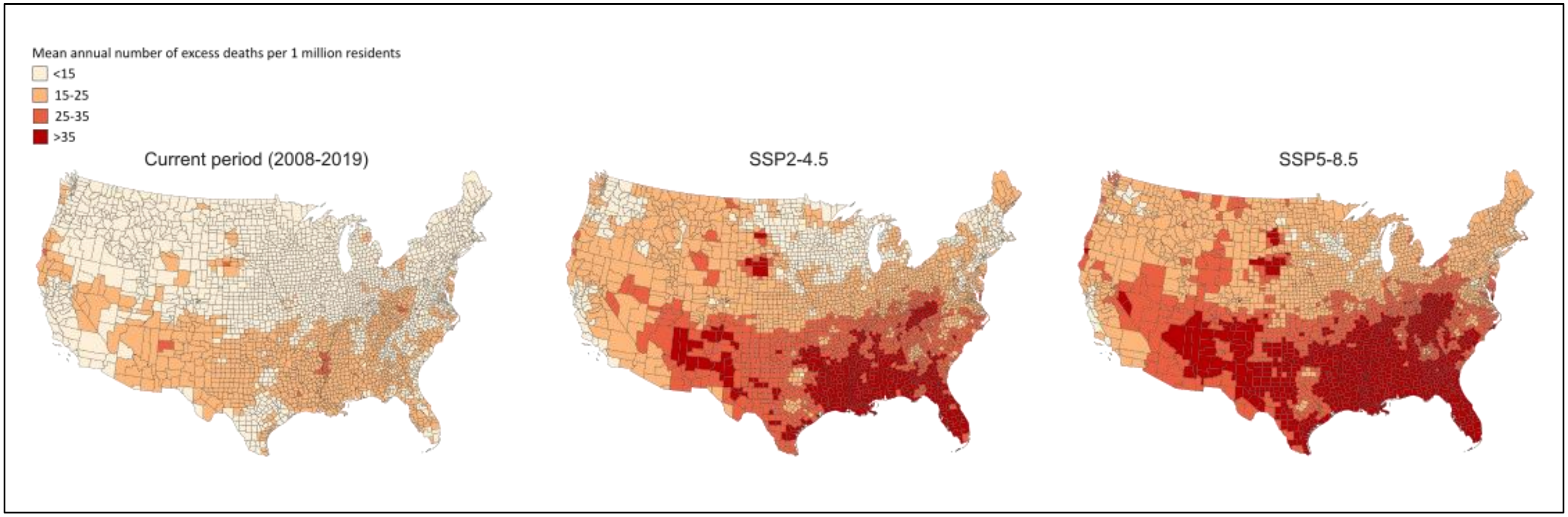

* Extreme heat defined as any day with mean temperature >97.5th percentile of historical (1979-2000) daily values for the county. Extreme cold defined as any day with mean temperature <2.5th percentile of historical (1979-2000) daily values for the county.

Estimated excess deaths based on Poisson fixed effects model with monthly and annual covariates from the 2008-2019 period (eMethods 7). Excess deaths were then estimated by calculating the difference between the number of predicted deaths in each county with all covariates at their observed value and the number of predicted deaths if there were no extreme heat days. For the projected number of excess deaths in the mid-century period, the number of extreme temperature days (hot and cold) and county population were replaced with projected values when calculating the difference while keeping the regression coefficients the same.

SSP - Shared Socioeconomic Pathways. RCP - Representative Concentration Pathway. SSP2-4.5 refers to a "Middle of the road" scenario for socio-economic changes and a lower increase in greenhouse gas emissions. SSP5-8.5 refers to a "Fossil-Fueled Development" scenario for socio-economic changes and a larger increase in greenhouse gas emissions.

© 2024 Khatana SAM et al. *JAMA Network Open*

eFigure 3 – Estimated mean annual extreme temperature associated excess deaths per 1 million adult residents in the current period (2008-2019) and mid-century (2036-2065) projections for females*

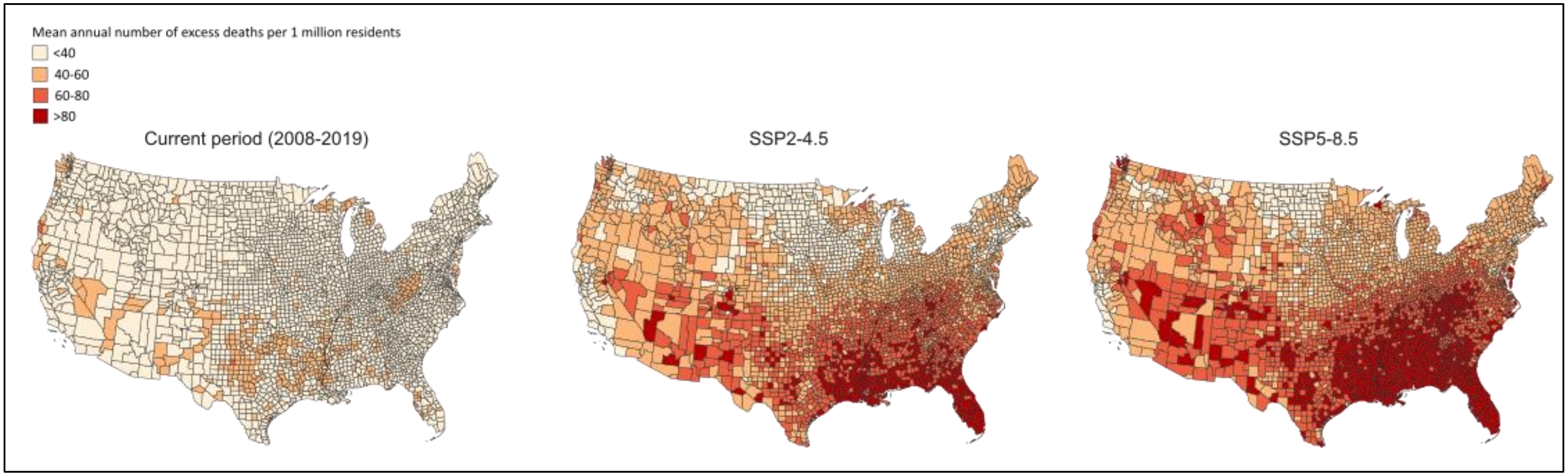

* Extreme heat defined as any day with mean temperature >97.5th percentile of historical (1979-2000) daily values for the county. Extreme cold defined as any day with mean temperature <2.5th percentile of historical (1979-2000) daily values for the county.

Estimated excess deaths based on Poisson fixed effects model with monthly and annual covariates from the 2008-2019 period (eMethods 7). Excess deaths were then estimated by calculating the difference between the number of predicted deaths in each county with all covariates at their observed value and the number of predicted deaths if there were no extreme heat days. For the projected number of excess deaths in the mid-century period, the number of extreme temperature days (hot and cold) and county population were replaced with projected values when calculating the difference while keeping the regression coefficients the same.

SSP - Shared Socioeconomic Pathways. RCP - Representative Concentration Pathway. SSP2-4.5 refers to a "Middle of the road" scenario for socio-economic changes and a lower increase in greenhouse gas emissions. SSP5-8.5 refers to a "Fossil-Fueled Development" scenario for socio-economic changes and a larger increase in greenhouse gas emissions.

© 2024 Khatana SAM et al. *JAMA Network Open*

eFigure 4 – Estimated mean annual extreme temperature associated excess deaths per 1 million adult residents in the current period (2008-2019) and mid-century (2036-2065) projections for males*

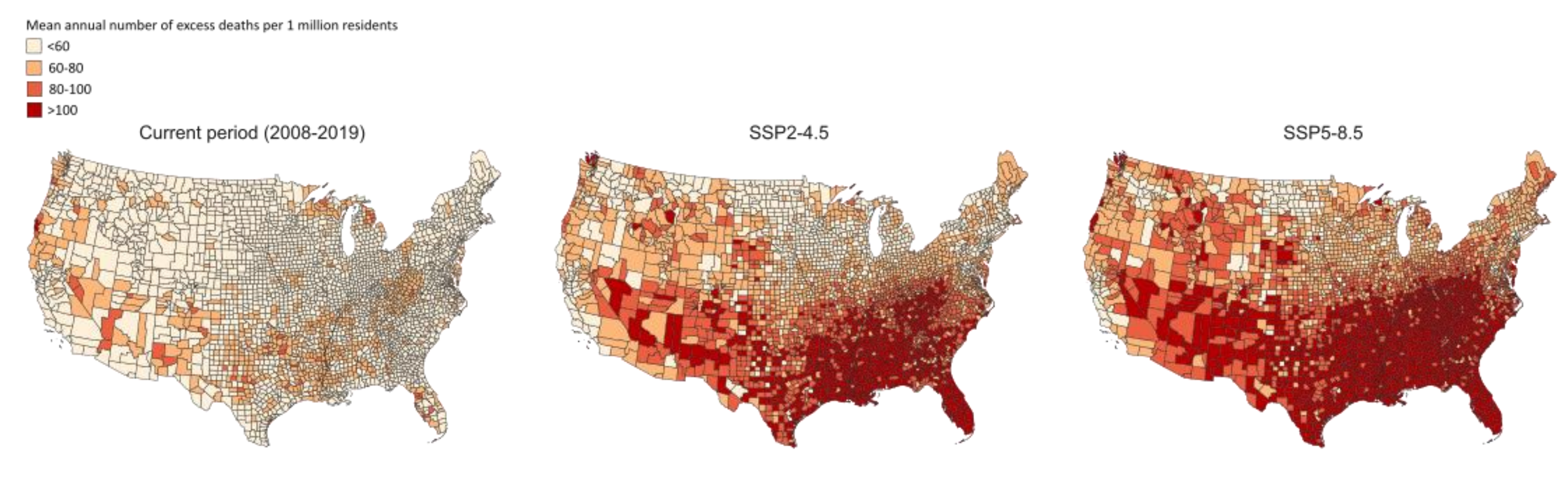

* Extreme heat defined as any day with mean temperature >97.5th percentile of historical (1979-2000) daily values for the county. Extreme cold defined as any day with mean temperature <2.5th percentile of historical (1979-2000) daily values for the county.

Estimated excess deaths based on Poisson fixed effects model with monthly and annual covariates from the 2008-2019 period (eMethods 7). Excess deaths were then estimated by calculating the difference between the number of predicted deaths in each county with all covariates at their observed value and the number of predicted deaths if there were no extreme heat days. For the projected number of excess deaths in the mid-century period, the number of extreme temperature days (hot and cold) and county population were replaced with projected values when calculating the difference while keeping the regression coefficients the same.

SSP - Shared Socioeconomic Pathways. RCP - Representative Concentration Pathway. SSP2-4.5 refers to a “Middle of the road” scenario for socio-economic changes and a lower increase in greenhouse gas emissions. SSP5-8.5 refers to a “Fossil-Fueled Development” scenario for socio-economic changes and a larger increase in greenhouse gas emissions.

© 2024 Khatana SAM et al. *JAMA Network Open*

eReferences

1. gridMET. (Accessed January 30, 2024, at https://www.climatologylab.org/gridmet.html.)

2. Daly C. Guidelines for assessing the suitability of spatial climate data sets. International Journal of Climatology: A Journal of the Royal Meteorological Society 2006;26:707-21.

3. Abatzoglou JT. Development of gridded surface meteorological data for ecological applications and modelling. International Journal of Climatology 2013;33:121-31.

4. Climate Data Operators. (Accessed December 1, 2024, at https://code.mpimet.mpg.de/projects/cdo.)

5. NCO User Guide. (Accessed December 1, 2023, at https://nco.sourceforge.net/nco.html.)

6. Dahl K, Licker R, Abatzoglou JT, Declet-Barreto J. Increased frequency of and population exposure to extreme heat index days in the United States during the 21st century. Environmental Research Communications 2019;1:075002.

7. Rothfusz LP, Headquarters NSR. The heat index equation (or, more than you ever wanted to know about heat index). Fort Worth, Texas: National Oceanic and Atmospheric Administration, National Weather Service, Office of Meteorology 1990;9023:640.

8. The Heat Index Equation. National Weather Service. (Accessed June 3, 2023, at https://www.wpc.ncep.noaa.gov/html/heatindex_equation.shtml.)

9. Abatzoglou JT, Brown TJ. A comparison of statistical downscaling methods suited for wildfire applications. International journal of climatology 2012;32:772-80.

10. Climate Modeling. (Accessed February 1, 2024, at https://www.gfdl.noaa.gov/climate-modeling/.)

11. Basics of Global Climate Models. (Accessed February 1, 2024, at https://www.climatehubs.usda.gov/hubs/northwest/topic/basics-global-climate-models.)

© 2024 Khatana SAM et al. *JAMA Network Open*

12. Tang J, Niu X, Wang S, Gao H, Wang X, Wu J. Statistical downscaling and dynamical downscaling of regional climate in China: Present climate evaluations and future climate projections. Journal of Geophysical Research: Atmospheres 2016;121:2110-29.

13. Joyce LA, Coulson D. Climate scenarios and projections: U.S. Department of Agriculture, Forest Service, Rocky Mountain Research Station; 2020 2020/05.

14. O'Neill BC, Carter TR, Ebi K, et al. Achievements and needs for the climate change scenario framework. Nat Clim Chang 2020;10:1074-84.

15. Riahi K, van Vuuren DP, Kriegler E, et al. The Shared Socioeconomic Pathways and their energy, land use, and greenhouse gas emissions implications: An overview. Global Environ Chang 2017;42:153-68.

16. van Vuuren DP, Edmonds J, Kainuma M, et al. The representative concentration pathways: an overview. Climatic Change 2011;109:5-31.

17. Intergovernmental Panel On Climate Change. Climate change 2014: synthesis report. Contribution of Working Groups I, II and III to the fifth assessment report of the Intergovernmental Panel on Climate Change. Core Writing Team, Pachauri, RK and Meyer, LA (eds) IPCC, Geneva 2014;151.

18. Lee J-Y, Marotzke J, Bala G, et al. Future global climate: scenario-based projections and near-term information. In: Masson-Delmotte V, Zhai P, Pirani A, et al., eds. Climate Change 2021: The Physical Science Basis Contribution of Working Group I to the Sixth : Assessment Report of the Intergovernmental Panel on Climate Change : Chapter 4. Genf, Switzerland: IPCC; 2021:1-195.

19. Explainer: How 'shared socioeconomic pathways' explore future climate change. Carbon Brief, 2018. (Accessed June 1, 2023, at https://www.carbonbrief.org/explainer-how-shared-socioeconomic-pathways-explore-future-climate-change/.)

20. Kc S, Lutz W. The human core of the shared socioeconomic pathways: Population scenarios by age, sex and level of education for all countries to 2100. Glob Environ Change 2017;42:181-92.

© 2024 Khatana SAM et al. *JAMA Network Open*

21. Lee JY, Marotzk J, Bala G, et al. The Physical Science Basis. Contribution of Working Group I to the Sixth Assessment Report of the Intergovernmental Panel on Climate Change Cambridge University Press Cambridge, UK; 2021.

22. Hauer ME. Population projections for U.S. counties by age, sex, and race controlled to shared socioeconomic pathway. Scientific Data 2019;6:190005.

23. Population Projections 2004 - 2030 by State, Age and Sex: Methodology Summary (Accessed June 5, 2023, at https://wonder.cdc.gov/wonder/help/populations/population-projections/methodology.html.)

24. Hamilton CH, Perry J. A short method for projecting population by age from one decennial census to another. Social Forces 1962;41:163-70.

© 2024 Khatana SAM et al. *JAMA Network Open*
