# Supplementary material for: Projections of Extreme Temperature–Related Deaths in the US
Source: JAMA Netw Open. 2024 Sep 20;7(9):e2434942. doi: 10.1001/jamanetworkopen.2024.34942 (PMC11415784; doi:10.1001/jamanetworkopen.2024.34942)
Supplement: Supplement 2. — Data Sharing Statement [file jamanetwopen-e2434942-s002.pdf]

Data Sharing Statement

Khatana. Projections of Extreme Temperature–Related Deaths in the US. *JAMA Netw Open*. Published September 20, 2024. doi:10.1001/jamanetworkopen.2024.34942

Data
Data available: No

Additional Information
Explanation for why data not available: Individual level mortality data obtained from the National Center for Health Statistics under agreement and cannot be shared by the authors.
